# Supplementary material for: Multimodality and the origin of a novel communication system in face-to-face interaction
Source: R Soc Open Sci. 2020 Jan 15;7(1):182056. doi: 10.1098/rsos.182056 (PMC7029942; doi:10.1098/rsos.182056)
Supplement: See the attached file for 6 titles [file rsos182056supp1.zip › SupportingMaterials/S1_InterpretationsOfFayEtAl.pdf]

Interpretations of Fay et al. (2013, 2014)

Fay et al. (2013, 2014)'s experiments find that multimodal signals provide no advantage over gestures alone. The authors conclude that this demonstrates the power of *motivated signs* rather than gestures per se. However, this has been interpreted in a variety of ways.

We used Google Scholar to find citations of Fay et al. (2014). We looked up the first 30 papers listed (excluding some that were not primarily about language) and located the passages in which they describe the results. Many of these also refer to Fay et al. (2013). Some papers only refer to the apparent superiority of gesture over vocalisaiton, while others refer to the point about iconic signs being more effective than arbitrary signs. Several include both points, and some suggest that the experimental evidence supports motivated signs being easier to make in the gestural modality (something that wasn't directly measured).

--

Perlman, M., Dale, R., & Lupyan, G. (2015). Iconicity can ground the creation of vocal symbols. Royal Society open science, 2(8), 150152.

"Given these basic findings in gesture and graphic media, an interesting question is the extent to which people can also generate vocal communication systems by this same process of iconic creation. On the face of it, many scholars have argued that they cannot [12,19,26–29]. Their arguments typically contend that gestures are special in their potential for iconicity, particularly when compared to vocalizations, because of the spatial nature of gestures and their resemblance to manual actions used for manipulating objects."

12 Armstrong DF, Wilcox SE. 2007 The gestural origin of language, 1 edn. Oxford, UK: Oxford University Press. Crossref, Google Scholar
19 Fay N, Arbib M, Garrod S. 2013 How to bootstrap a human communication system. Cogn. Sci. 37, 1356–1367.
26 Arbib MA. 2005 From monkey-like action recognition to human language: an evolutionary framework for neurolinguistics. Behav. Brain Sci. 28, 105–124. (doi:10.1017/S0140525X05000038)
27 Corballis MC. 2003 From hand to mouth: the origins of language. Princeton, NJ: Princeton University Press.
28 Tomasello M. 2008 Origins of human communication. Cambridge, MA: The MIT Press.
29 Hockett CF. 1978In search of Jove's brow. Am. Speech 53, 243–313. (doi:10.2307/455140)

--

Goldin-Meadow, S., & Brentari, D. (2017). Gesture, sign, and language: The coming of age of sign language and gesture studies. Behavioral and Brain Sciences, 40.

"and it is commonly claimed that the manual modality offers greater potential for iconicity than the oral modality (see Fay et al. 2014 for experimental evidence for this claim). "

--

Tamariz, M., Ellison, T. M., Barr, D. J., & Fay, N. (2014). Cultural selection drives the evolution of human communication systems. Proceedings of the Royal Society B: Biological Sciences, 281(1788), 20140488.

"While the visual modality offers benefits for communication over the auditory modality [31,48],"
31: Fay N, Arbib M& Garrod S. 2013How to bootstrap a human communication system. Cogn. Sci. 37, 1356–1367.
48: Fay N, Lister CJ, Ellison TM& Goldin-Meadow S. 2014Creating a communication system from scratch: gesture beats vocalization hands down. Front. Psychol. 5, 354.

--

Perlman, M., & Cain, A. A. (2014). Iconicity in vocalization, comparisons with gesture, and implications for theories on the evolution of language. Gesture, 14(3), 320-350.

"In one set of experiments, participants played a communication game in which they used manual gestures, vocalizations, or a combination to communicate to a partner words from a shared list that included emotions, actions, and objects (Fay, Arbib, & Garrod, 2013; Fay, Lister, Ellison, & Goldin-Meadow, 2014). Players demonstrated a moderate amount of success in the vocal condition, but they were significantly better with gestures, and gained no added benefit from the combi-nation of modalities. The authors conclude that the iconic, motivated nature of gestures serves to ground the creation of labels, whereas this grounding is not af-forded by the more arbitrary nature of vocalizations."

--

Winter, B., Perlman, M., Perry, L. K., & Lupyan, G. (2017). Which words are most iconic?. Interaction Studies, 18(3), 443-464.

"Experimental studies show that people can take advantage of iconicity to improvise novel, meaningful vocal-izations (Fay, Arbib, & Garrod, 2013; Perlman, Dale, & Lupyan, 2015),"

--

Żywiczyński, P., Wacewicz, S., & Sibierska, M. (2018). Defining pantomime for language evolution research. Topoi, 37(2), 307-318.

"The dominance of the visuomotor channel in pantomime is dictated by its iconic potential, superior to the vocal-auditory channel in the context of iconically bootstrapping a communication system (see e.g. Fay et al. 2013, 2014). However, mimetic communication is not at all constrained to this one channel but makes active and frequent use of other modalities and semiotic resources—“facial expression and other modes of emotional expression, such as a variety of calls and cries, and strictly prosodic aspects of voice modulation would also have fitted into a purely mimetic culture” (Donald 1991: 78)."

--

Roberts, G., Lewandowski, J., & Galantucci, B. (2015). How communication changes when we cannot mime the world: Experimental evidence for the effect of iconicity on combinatoriality. Cognition, 141, 52-66.

"Iconic forms provide “scaffolding for the cognitive system to connect linguistic form and embodied experience” (Perniss, Thompson, & Vigliocco, 2010, p. 12), making it easier to establish new signs (Fay et al., 2013, Fay et al., 2014)."

--

Fay, N., Ellison, M., & Garrod, S. (2014). Iconicity: From sign to system in human communication and language. Pragmatics & Cognition, 22(2), 244-263.

"Would non-linguistic vocalization, a mostly symbolic communication medium, be as successful as gesture? If so, this would argue against the importance of motivated signs when bootstrapping human communication. This was tested in a pair of referential communication studies in which human par-ticipants tried to communicate a set of recurring concepts (various emotions, ac-tions and object items) to a partner using only gesture, non-linguistic vocalization or a combination of gesture and vocalization (Fay, Arbib, & Garrod, 2013; Fay, Lister, Ellison, & Goldin-Meadow, 2014). Communication success was higher for gesture than non-linguistic vocalization in both studies, and gesture was either as effective as the combined modalities (Fay et al., 2013) or was more effective (Fay et al., 2014). While these findings indicate the superiority of gesture over non-linguistic vo-calization when creating a communication system for scratch, they do not rule out a role for non-linguistic vocalization in the earliest stages of language evolution."

Coppola, M., & Brentari, D. (2014). From iconic handshapes to grammatical contrasts: longitudinal evidence from a child homesigner. Frontiers in psychology, 5, 830.

"In the realm of experimental semiotics, Fay et al. (2013, 2014) argue that gesture is likely to bootstrap human communication systems in the absence of linguistic input precisely because it affords greater iconicity than the auditory modality.
...
In the larger semiotic context of iconicity, Fay et al. (2013, 2014) have proposed that the greater degree of iconicity afforded by the visuo-gestural modality (vs. the auditory-aural modality) allows faster and more efficient development of human communication systems in the absence of language input. This may be true for human communication, broadly construed, but the present results would suggest that while iconicity is clearly available in the visual realm, its use during the creation of a sign language is much more complicated than its wholesale exploitation. "

Goldin-Meadow, S. (2017). What the hands can tell us about language emergence. Psychonomic bulletin & review, 24(1), 213-218.

"I consider the possibility that the oral modality took over segmented and combinatorial encoding, not because of its strength at conveying information in a segmented and combinatorial format, but because of its weakness in conveying information mimetically. ... As an example, if adults are asked to create labels for objects and actions with their hands and no voice, they create comprehensible symbols more easily than if they are asked to create the same labels with voice alone (Fay, Lister, Ellison & Goldin-Meadow, 2014).

Conveying information in an analog and mimetic format turns out to be important to human communication, and this function is well served by the manual modality—in the form of spontaneous gestures that accompany speech (Feyereison & de Lannoy, 1991; McNeill, 1992). If both segmented and mimetic encoding are essential to human communication, and if mimetic encoding falls to the manual modality because it's so good at it, segmented encoding falls, by default, to the oral modality. This argument rests on (at least) two assumptions: that the manual modality is good at segmented and combinatorial encoding and that mimetic encoding is important to human communication and is handled well by the manual modality. "

Little, H., Eryılmaz, K., & de Boer, B. (2017). Signal dimensionality and the emergence of combinatorial structure. Cognition, 168, 1-15.

"Although it is hard to quantify pre-cisely, iconic structure is more prevalent in sign language than inspoken language. This assumption is supported by experimentalevidence demonstrating that it is more difficult to be iconic usingvocalisations than it is with gestures (Fay, Lister, Ellison, &Goldin-Meadow, 2014)."

Eryilmaz, K., & Little, H. (2017). Using leap motion to investigate the emergence of structure in speech and language. Behavior research methods, 49(5), 1748-1768.

"Further, different levels of iconicity are possible using different linguistic modalities (Fay et al. 2014),"

Hall, M. L., Ahn, Y. D., Mayberry, R. I., & Ferreira, V. S. (2015). Production and comprehension show divergent constituent order preferences: Evidence from elicited pantomime. Journal of memory and language, 81, 16-33.

"We ultimately argue that pantomimic gesture can even reveal factors that influence the structure of spoken languages.
We are not the first to recognize the value of studying pantomimic gesture (sometimes called “silent gesture”; henceforth, “elicited pantomime”); a number of other researchers have also used elicited pantomime to probe various features of human communication (Fay et al., 2013, 2014; ..."

Sandler, W. (2017). The challenge of sign language phonology. Annual review of Linguistics, 3, 43-63.

"other experiments show that it is much more difficult to create correspondence between form and meaning with auditory than with visual signals (Fay et al. 2015). As a result, such phenomena in spoken languages are much less widespread than in sign languages, and the relation between form and meaning is less direct and more language specific"

Galantucci, B. (2017). Experimental semiotics. In Oxford Research Encyclopedia of Linguistics.

"Four ES studies provide experimental evidence that people tend to rely on motivated signs because these signs facilitate the bootstrapping of a communication system (Fay et al., 2013, 2014"

--

Perlman, M., & Lupyan, G. (2018). People can create iconic vocalizations to communicate various meanings to naïve listeners. Scientific reports, 8(1), 2634.

"Many theories of language evolution have assumed that people have very limited means to express meanings using novel (i.e., not already conventionalized) vocalizations. As a result, it is commonly posited that manual gestures must have played an essential role in bootstrapping the formation of spoken symbols1,3,4,13,14. However, evidence from spoken languages16,17, as well as from experiments41,43, suggests that people are surprisingly adept in inventing and interpreting novel vocalizations via iconicity – resemblance between form and meaning.

... Other studies have shown that these invented vocalizations are, to some degree, understandable to naïve listeners. One experiment compared the use of non-linguistic vocalization and gesture to communicate 18 items that included emotions (e.g. disgust, tired), actions (e.g. throwing, chasing) and objects (e.g. predator, tree)41,42."

41. Fay, N., Arbib, M. & Garrod, S. How to Bootstrap a Human Communication System. Cogn. Sci. 37, 1356–1367 (2013).

42. Fay, N., Lister, C. J., Ellison, T. M. & Goldin-Meadow, S. Creating a communication system from scratch: gesture beats vocalization hands down. Front. Psychol. 5, (2014).

Berent, I., & Goldin-Meadow, S. (2015). Language by mouth and by hand. Frontiers in psychology, 6, 78.

"In contrast, when adult speakers are engaged in a communication game, Fay et al. (2014) find a strong advantage for gestures over speech (alone, or even in combination with gesture)—a finding that the authors attribute to the affordance of the manual modality for iconicity. The gesture advantage in adult speakers does not speak directly to language evolution in humans, but the results are in line with the possibility that proto-language was gestural. "

Lupyan, G., & Winter, B. (2018). Language is more abstract than you think, or, why aren't languages more iconic?. Philosophical Transactions of the Royal Society B: Biological Sciences, 373(1752), 20170137.

"People also have aknack for creating iconic gestures [104,105] and vocalizations[106,107], and for understanding gestures and vocalizationscreated by others to express a wide range of meanings. Suchadvantages of iconic forms had led some to argue that iconicity played a key role in the evolution of language (e.g. [108])."

104. Fay N, Arbib M, Garrod S. 2013 How to bootstrap ahuman communication system.Cogn. Sci.37,1356–1367. (doi:10.1111/cogs.12048)
105. Fay N, Lister CJ, Ellison TM, Goldin-Meadow S. 2014Creating a communication system from scratch:gesture beats vocalization hands down.Front.Psychol.5, 354. (doi:10.3389/fpsyg.2014.00354)
108. Imai M, Kita S. 2014 The sound symbolismbootstrapping hypothesis for language acquisitionand language evolution.Phil. Trans. R. Soc. B369,20130298.

Zlatev, J., Wacewicz, S., Zywiczynski, P., & van de Weijer, J. (2017). Multimodal-first or pantomime-first?. Interaction Studies, 18(3), 465-488.

"Two recent studies (Fay, Arbib, & Garrod, 2013; Fay, Lister, Ellison, & Goldin-Meadow, 2014) used the method known as a “referential game” ... gesture/pantomime with or without vocalization had a clear advantage, leading the authors toconclude that “gesture outperforms non-linguistic vocalization because it lends itself more naturally to the production of motivated [i.e. iconic] signs” (Fay, Arbib, & Garrod,2013: 1). Interestingly, the multimodal condition of gesture combined with vocalizationdid not result in greater communicative success than silent gesture in both experiments;In one case, communicating Action items in

the multimodal condition was slightly but significantly less effective than silent gesture alone (Fay, Lister, Ellison, & Goldin-Meadow, 2014)."

Meier, R. (2016, March 07). Sign Language Acquisition. Oxford Handbooks Online. Ed. Retrieved 15 Mar. 2019, from http://www.oxfordhandbooks.com/view/10.1093/oxfordhb/9780199935345.001.0001/oxfordhb-9780199935345-e-19.

"Without access to the resources for iconic representation that are available in the visual-gestural modality, it is hard to imagine how these children could have developed an effective vocabulary; see Fay, Lister, Ellison, and Goldin-Meadow (2014) for an experimental probe of the efficacy of vocalization versus gesture in adult innovation of a communication system in a laboratory situation."

Perlman, M. (2017). Debunking two myths against vocal origins of language. Interaction Studies, 18(3), 376-401.

"The second negative claimis that, unlike gestures, vocalizations lack any substantial potential to ground meaning throughiconicity,orresemblance between form and meaning. ... The critical need to establish a connection between symbol and meaning motivatesthe theory that iconic (and indexical) gestures arenecessary to bootstrap the creation of an arbitraryvocal symbol system(Armstrong & Wilcox, 2007; Fay, Arbib, & Garrod, 2013; Fay, Lister, Ellison, & Goldin-Meadow, 2014).

...

It follows from this reasoning that vocalizations must have been “piggybacked” (Tomasello, 2008, p. 330) or “boot-strapped” (Fay et al., 2013)on gestures because of their “vastly greater possibility for iconic productivity in the visual medium” (Armstrong & Wilcox, 2007, p. 123).

...

The main conclusion of the studies [Fay, Arbib, & Garrod, 2013; Fay, Lister, Ellison, & Goldin-Meadow, 2014] was that participants performed better with gestures than vocalizations. However,players showed success with vocalizations, particularly for emotions and actions, which were identifiedat accuracy levels that well exceeded chance.

...

There has been a tendency in previous scholarship on language evolution to pit gestures against vocalizations, with an emphasis on determining which came first. For instance, Fay et al. (2014) highlighted their comparison of modalities with the article title “Gesture beats vocalization hands down.” Yet,while I have set out here to dispose of the main arguments against vocal origins, this does not detract from the positive arguments in favor of gestures playing a role in the origins of language. The gesturing of great apes is impressively flexible, and the iconic potential of gestures is well attested(e.g. Arbib, Liebal, & Pika, 2008; Cartmill, Beilock, & Goldin-Meadow, 2012). Thus, the current weight of evidencesupports the

hypothesisthat languagebegan through iconic communication coordinated across both the vocal and gestural modalities.
Notably, this is a different claim than some previous proposalsfor multimodal originsof language. Some gesture scholarshave argued for a multimodal evolution of language in the context ofatheory of human communication in which gestures mainly incorporate the imagistic or iconic aspects of expression, while vocalizations serve mainly for the conventionalized, linguistic channel (e.g. Kendon, 2014; McNeill 2012)."

Lister, C. J., & Fay, N. (2017). How to create a human communication system. Interaction Studies, 18(3), 314-329.

"Compared to vocal communication, gesture more naturally lends itself to the production of motivated signs. For example, it is easier to imagine how to cre-ate a motivated sign for the meanings ‘running’, ‘tired’ or ‘apple’ by gesture than by non-lexical vocalization (making sounds that are not words). If correct, it fol-lows that gesture will be a better means of bootstrapping a human communication system compared to non-lexical vocalization. This was tested by Fay etal. (2013, 2014). They compared communication in these two modalities, and predicted that participants would be more successful at bootstrapping a novel communication system through gesture than through vocalization. They had participants play a ‘charades game’ that prohibited the use of the participants’ pre-existing language, limiting communication to gestures or non-lexical vocalizations. Their results confirmed the hypothesis: participants who gestured were more successful at com-municating a range of different meanings (emotion, action and object words) to a partner, compared to those who relied solely on vocalizations. These findings suggest that motivated signs help new communication systems get started because they facilitate mutual understanding (i.e., cognitive alignment).
Although Fay etal. (2013, 2014) demonstrated a benefit of gesture over non-lexical vocalization for bootstrapping human communication, they did not mea-sure sign motivation.
"

Lister, C. J., Fay, N., Ellison, T. M., & Ohan, J. (2015). Creating a new communication system: Gesture has the upper hand. In Proceedings of the 37th Annual Meeting of the Cognitive Science Society.

"In line with the proto-sign account, participants who gestured were more successful at communicating meanings to their partner than the participants who were restricted to the vocal modality. ... Fay et al. (2014) suggested that gesture was a more successful mode of communication compared to vocalization because it more naturally lends itself to the production of ‘motivated’ signs (i.e. iconic or indexical signs that share a direct, or non-arbitrary, relationship with their referent). While the authors did not directly examine sign motivation, they suggested that participants who gestured were better able to physically represent the concepts they wished to communicate (e.g., through mimicry or pantomime)."

Little, H., Perlman, M., & Eryilmaz, K. (2017). Repeated interactions can lead to more iconic signals. In the 39th Annual Conference of the Cognitive Science Society (CogSci 2017) (pp. 760-765). Cognitive Science Society.

"One hypothesis is that the first words were created using iconicity(Fay, Ellison, & Garrod, 2014; Imai & Kita,2014; Perlman, Dale, & Lupyan, 2015; Perniss, Thompson,& Vigliocco, 2010).
...
It is widely assumed that spoken languages have markedly less iconicity than signed languages. Yet, it is unclear why this is the case. One widely argued reason is that the vocal-auditory modality affords little iconicity to represent a rich array of meanings (Armstrong & Wilcox, 2007). This argument is supported mainly by comparing impressions of the iconicity ofgesture and sign with vocalisations and speech, and also by experimental studies finding that gestures were more effective than non-linguistic vocalisations at communicating different meanings (Fay, Arbib, & Garrod, 2013; Fay, Lister,Ellison, & Goldin-Meadow, 2014). "

Perlman, M., Little, H., Thompson, B., & Thompson, R. L. (2018). Iconicity in Signed and Spoken Vocabulary: A Comparison Between American Sign Language, British Sign Language, English, and Spanish. Frontiers in psychology, 9, 1433.

"We suggest that part of the reason for the previous lack of detailed comparative studies between modalities is the widespread assumption that signed languages are far more iconic than spoken languages ... This idea figures prominently in many theories of language evolution that argue that the first symbolic forms must have been built from gestures (e.g., Corballis, 2003; Armstrong and Wilcox, 2007; Tomasello, 2008; Arbib, 2012; Fay et al., 2014). These gesture-first theories depend critically on the premise that signs afford much more iconicity than words."
