# Supplementary material for: Multimodality and the origin of a novel communication system in face-to-face interaction
Source: R Soc Open Sci. 2020 Jan 15;7(1):182056. doi: 10.1098/rsos.182056 (PMC7029942; doi:10.1098/rsos.182056)
Supplement: See the attached file for 6 titles [file rsos182056supp1.zip › SupportingMaterials/S2_Gallery.html]

 
 
 
 
 
 
 Gestures for visual stimuli 
 The examples below have some amount of systematicity. Shapes that are mostly 'filled' (shapes 3,4 and 5) are communicated by moulding a circular outline and 'filling it in' with a fist.   
 
  Num  Stimulus  Response  Description  
  1       The director pokes the air to establish a 'spoke' then uses their index finger to trace lines at different angles around this spoke.  
  2       Director produces small circular motions with both her index fingers, as if tracing small circles.  
  3       Director raises her right hand, followed by her left hand, both roughly to the level of the eyes. The hands forms a circle in front of her eyes (3a). The director then clenches her left hand into a fist (3b), and bangs it close to her right hand. Next, she uses her left index finger to trace the outer contours of her right hand, which is still shaped as a half circle (3c).  
  4       Director raises both her hands, roughly to the level of the eyes. The hands forms a circle in front of her eyes (4a). The director then clenches her left hand into a fist, and bangs it close to her right hand (4b). Next, she uses her left index finger to pinpoint several dots in the air (4c), close to her right hand. The dots may relate to the small spaces between lines in the image.  
  5       Director raises both her hands, roughly to the level of the eyes. The hands forms a circle in front of her eyes (5a). The director then clenches her left hand into a fist, and bangs it close to her right hand (5b). Next, she uses both her index fingers to pinpoint several dots in the air (5c), at the level of her forehead. After an inquistive gaze from her partner, she again shapes a circle with both her hands, followed by the fist-clenching and banging sequence. The director then uses her left index finger to trace several lines in the air (5d, 5e), close to her right hand, which is still shaped as a half circle.  
  6       Director raises both her hands and molds a triangle using her thumbs and index fingers, to refer to the triangular shapes within the image.  
  7       Director raises both her hands, the left hand almost perpendicular to the table, palm facing outward, the right hand almost touching the left hand, forming a 90 deegres angle (7). The left hand has the index and middle finger extended and the right hand has the index, middle and ring finger extended.  The parallel fingers mimic the longest parallel lines in the image.  
  8       The director uses her left index finger to trace several lines in the air (8a, 8b). There are sets of parallel lines which mimic the parallel lines in the image. The rapid tracing of several lines may relate to the additional complexity of the image compared to image 7.  

 
  
* Participants gave permission to use their images, but we provide some anonymity anyway.
  
 Gestures for auditory stimuli 
 
  Num  Stimulus  Response  Description  
  1        {Clenched fist} Director moves right hand horizontally, away from her body, as if pulling a handle/ lever or opening a door. She moves the torso in the same direction, up until the arm is fully stretched out.  
  2        {Open hand} Director traces a large circle in the air around her head (2a). She then lowers both her arms down in parallel, in a straight motion and then spreading out, waggling her fingers (2b). The sequence is repeated twice.  
  3        {Open hand, outstretched fingers} Director slowly brings both her arms down, from above her head up until roughly her lower legs. The movement is repeated twice.  
  4        Director shapes hands as if handling a gun/ rifle. She thens pulls the front hand down, as if having pulled the trigger.  
  5        {Curved fingers, outstreched fingers} Director slides her left hand horizontally, away from her body, she then moves the hand down, as if pulling something down (5a). This movement is completed quickly, mimicking the short length of the audio sequence. The sequence is repeated 3 times.

{Open hand, outstretched fingers} Director brings both hands down, from above her head up until her upper chest (5b). She then moves each arm in an opposite direction, hands flat down, facing the table.  
  6        Director traces a circle around the upper left-hand area of her chest, where the heart is, using both her index fingers (6a). Then, using her right hand, flat open, she produces a repeated beating gesture, the palm beating away from the traced area (6b, 6c). The beating heart relates to the pulsing rhythm in the audio sample.  
  7        {Open hand, outstretched fingers} Director brings both hands down, from above her head up until her upper chest (7a). She then moves each arm in an opposite direction, hands flat down, wobbling her fingers in the process (7b, 7c). The sequence is repeated twice. The wobbling may relate to the oscillation in the audio clip.  
  8        {Open hand, hand perpendicular to the table} Director moves her left hand away from her body, up until the arm is almost fully streched, in roughly 6 repeated movements/ increments.  

 
  
 Vocalisations for visual stimuli 
 This pair has created a system where high-pitched or 'hmm' sounds refer to lines.  Sequences of two of these sounds mimic parallel lines. /ʃplaʃ/ refers to 'filled in' shapes. 90% of this participant's vocal productions for images were guessed correctly. 
 The sample is repeated twice within each audio file. 
 In these samples you can also hear mouse clicks and the matcher indicating that they understand.  Note that the volume may be lower than for the stimuli files.  
 
  Num  Stimulus  Response  Description  
  1       The director produces several 'individual' high-pitched sounds, mimicking the individual lines in the image, and they are relatively higher than for stimuli 7, perhaps indicating the apparent weightlessness of the image (it may look like a dandelion seed or ball of fluff).  
  2       The director uses an onomatopoeic representation of a spring to convey the spring-like circles in the image.  
  3       /ʃplaʃ/ refers to a 'filled-in' shape, with the word being similar to a conventionalised onomatopeia for water sounds (the shape somewhat resembling a splash of liquid).  
  4       The director produces /ʃplaʃ/ to refer to the 'filled-in' aspect of the shape, then adds a grating sound to relate the jagged shapes within the image.  
  5       The director produces /ʃplaʃ/ to refer to the 'filled-in' aspect of the shape, then adds high-pitched 'hmm' noises to indicate lines.  
  6       The director produces alternating high and low pitches, perhaps to indicate lines with angles.  
  7       The high-pitch 'hmm' sounds indicate lines.  
  8       The director produces alternating high-low-high pitches with several repetitions, perhaps to indicate many intersecting lines.  

 
  
 Vocalisations for auditory stimuli 
 Directors mostly try to mimic the sound directly. 
 The sample is repeated twice within each audio file. 
 
 
  Num  Stimulus  Response  
  1        
  2        
  3        
  4        
  5        
  6        
  7        
  8        

 
 
 
 
