# Supplementary material for: Multimodality and the origin of a novel communication system in face-to-face interaction
Source: R Soc Open Sci. 2020 Jan 15;7(1):182056. doi: 10.1098/rsos.182056 (PMC7029942; doi:10.1098/rsos.182056)
Supplement: See the attached file for 6 titles [file rsos182056supp1.zip › SupportingMaterials/S3_Multimodal_QualitativeSequences.pdf]

Sequences

The following is a step-by-step description of how members of dyad 18 described visual item 5 in all 8 trials in which it was presented to them as a target. There were four 'games' where each item appeared as a target once for participant 1 and once for participant 2 (in an unpredictable order with other stimuli). Note that we did not transcribe the matcher's sign for agreement (a closing 3rd turn, for which a conventional sign was provided by the experimenters), nor initial signs for attracting attention before the main sequence.

Visual item 5:

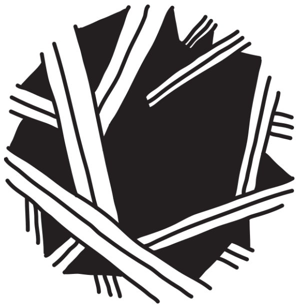

Coding conventions: 'P' - player; 'M' - moment; -//- - end of trial

Game 1

Several pairs divided the stimuli into predominantly “filled” and “unfilled” stimuli. P1 in this pair establishes a convention of pointing to their black t-shirt to refer to this category. Then they cross their fingers to represent the prominent crossing lines of figure 5 (compared to the other ‘filled’ images that do not have prominent internal lines).

P1:	M1 [traces circle on table]	[two hands] [index finger]
	M2 [taps chest]	[one hand] [open hand]
	M3 [places fingers on table]	[two hands] [index finger]
	M4 [traces on table]	[one hand]

-//- -

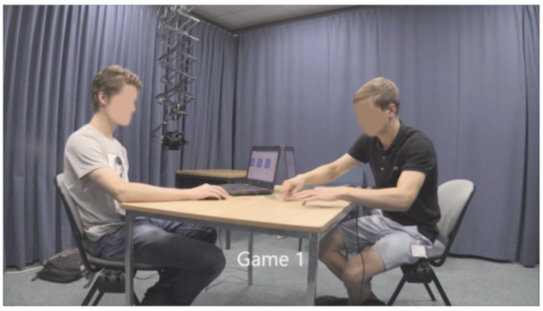

Fig.1: M1

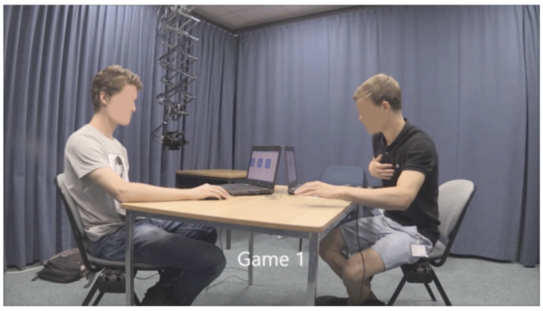

Fig.2: M2

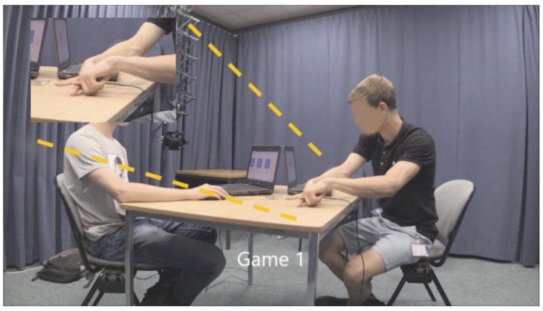

Fig.3: M3

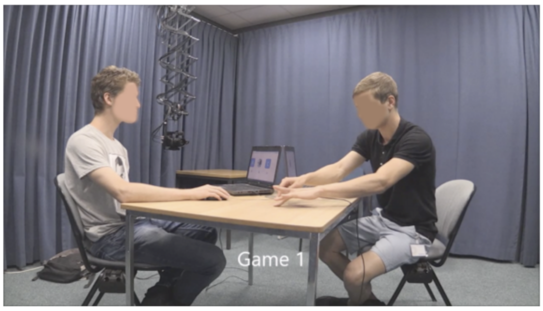

Fig.4: M4

Four moments within a trial in game 1. P1 appears on the right, P2 appears on the left.

As it happens, the very next trial also asked P2 to communicate visual stimulus 5. So P2 points at P1's shirt and copies their strategy of tracing the image on the table. P2 is pointing not only at the direct affordance of the t-shirt, but also back at the previous interaction.

P2:	M1 [points at partner's shirt]	[index finger]
	M2 [traces on table]	[two hands] [index finger]
	M3 [places fingers on table]	[two hands] [index & middle finger] [coupled fingers]

-//- -

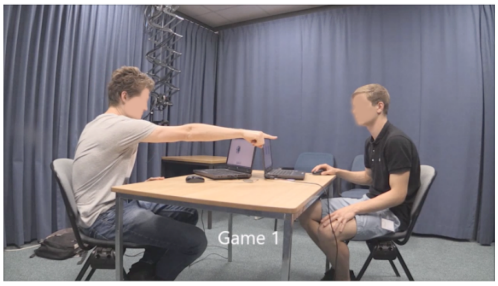

Fig.1: M1

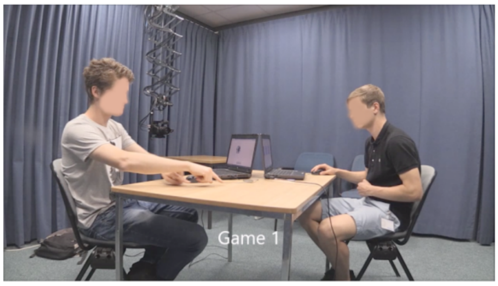

Fig.2: M2

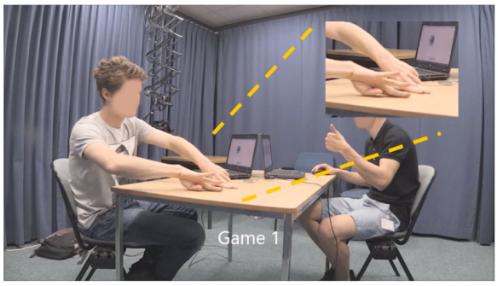

Fig.3: M3

Game 2

In the second game, the same strategy has become conventionalised, though P2 uses two fingers on each hand to form the second part of the sign.

P1:	M1 [grabs/ pulls shirt]	[one hand]
	M2 [places fingers on table]	[two hands] [index finger]

-//- -

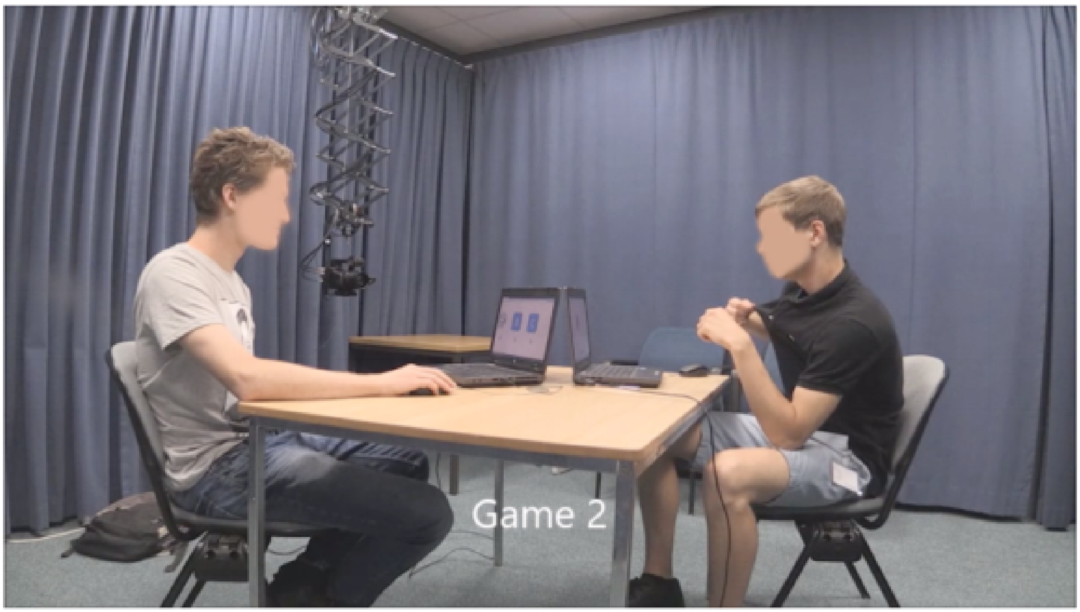

Fig.1: M1

P2:	M1 [points at partner's shirt]	[index finger]
	M2 [places fingers on table]	[two hands] [index & middle finger] [coupled fingers]
	M3 [points at his partner's shirt]	[index finger]

-//- -

Game 3

In game 3 there is some confusion, leading to an extended sequence of repair. P1 does the sign of crossing their fingers on the table, but does not point to their shirt first. P2 indicates they don't understand by shaking their head and vocalising. This is a way of initiating open repair. P1 repeats their signal (a typical response to open repair), but P2 still does not understand. P2 points at their own shirt, and then P1's shirt. By contrasting P2's white t-shirt (indicating 'unfilled' stimuli) with the established convention of P1's black t-shirt, P2 is effectively asking "do you mean the unfilled crossed-shape, or the filled crossed-shape?". This might be classified as candidate understandings, inviting P1 to confirm which they mean. In this sense, the response is more like what one would expect from a "restricted" open repair (compared to a simple confirmation), though technically P2's repair does not 'point to' part of P1's signal. Either way, this sequence of two repairs follows a classic pattern of 'upgrading' from a more open to a more restricted repair type.

P1:	M1 [places fingers on table]	[two hands] [index & middle finger] [coupled fingers]
	M2 [traces circle on table]	[two hands] [all fingers]
P2:	M3 [shakes head/ vocalizes]	[bilabial nasal sound]
P1:	M4 [places fingers on table]	[two hands] [index & middle finger] [coupled fingers]
P2:	M5 [points at own shirt/ vocalizes]	[index finger] [nasal vowel sound]
	M6 [points at partner's shirt/ vocalizes]	[index finger] [nasal vowel sound]
P1:	M6 [grabs/ waves shirt]	[one hand]
P2:	M7 [points at partner's shirt]	[index finger]
	M8 [points at own shirt/ vocalizes]	[index finger] [bilabial nasal sound]
P1:	M9 [grabs/ waves shirt]	[two hands]
	M10 [traces circle on table]	[two hands] [index finger]
	M11 [points at partner's shirt]	[index finger]
	M12 [places fingers on table]	[two hands] [index & middle finger][coupled fingers]

-//- -

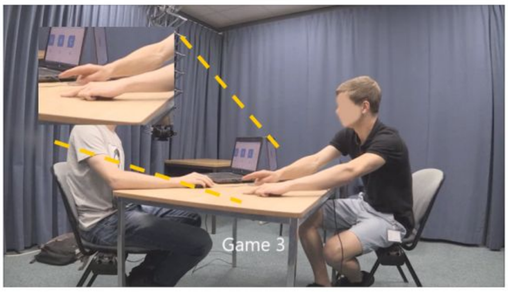

Fig.1: M1

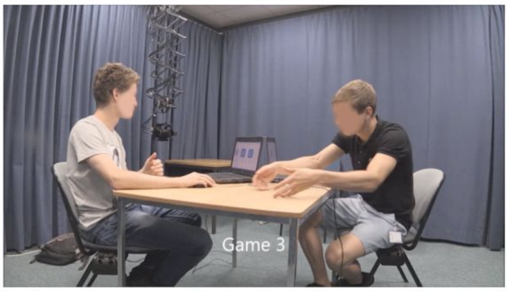

Fig.2: M2

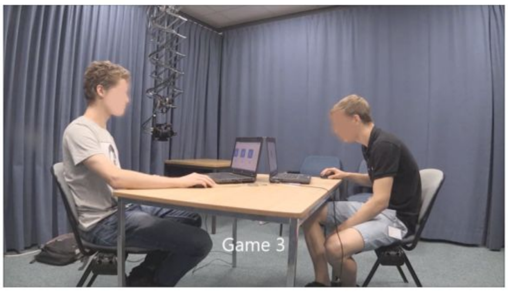

Fig.3: M3

When P2 is the director, they revert to the two-step sign (pointing at P1's shirt, placing fingers on table). Note that they also produce the sound that they used in the prior sequence during each step of the sign, perhaps to draw attention to the idea that both parts of the sign should be produced.

P2: M1 [points at partner's shirt/ vocalizes] [index finger] [bilabial nasal sound]
M2 [places fingers on table/ vocalizes] [two hands][index & middle finger][coupled fingers] [bilabial nasal sound]

-//- -

Game 4

In this game, some uncertainty leads the director (Player 1) and matcher (Player 2) to communicate extensively before the matcher finally makes a choice. The matcher produces several vocalizations while communicating gesturally, these vocalizations drawing the attention of the director to the visual displays he is producing. Aside from pragmatic vocalizations, the matcher also produces a referential acoustic display (moment 7), which seems to complement the reference being established by its visible gestural counterpart.

As with the previous game, P1 places crossed fingers on the table, but without the established convention (at least for P2) of pointing to their shirt. This leads to P2 initiating repair: they vocalise (perhaps to draw attention), and point at themselves. As above, this might be a candidate understanding, asking P1 to confirm whether it is an “unfilled” shape.

P1: M1 [places fingers on table] [two hands] [index & middle finger] [coupled fingers]

P2: M2 [points at own shirt/ vocalizes] [index finger][/ɐ:/]

P1: M3 [taps chest] [one hand] [open hand]

P2: M4 [points at partner’s shirt/ vocalizes] [index finger][/hɐ↗/ + /ɐ/]

P1: M4 [traces circle on table] [two hands] [index finger]

P2: M5 [points at partner’s shirt/ vocalizes] [index finger][/hɐ↗/]

M6 [places fingers on table/ vocalizes] [one hand] [all fingers] [finger tips][/muɔ:/]

M7 [places fingers on table] [two hands][index & middle finger] [coupled fingers][/hɐ↗/]

M8 [points at partner’s shirt] [index finger]

-//- -

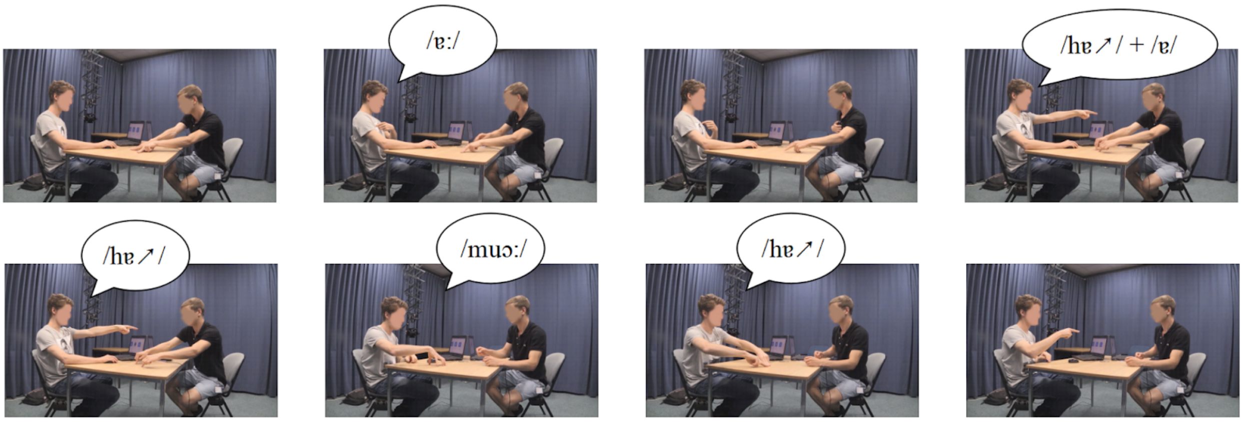

When P2 is the director, they revert to the conventional sign.

P2: M1 [points at partner’s shirt] [index finger]

M2 [traces circle on table/ vocalizes] [two hands] [index finger]

M3 [places fingers on table/ vocalizes] [two hands] [index & middle finger] [coupled fingers]

-//- -

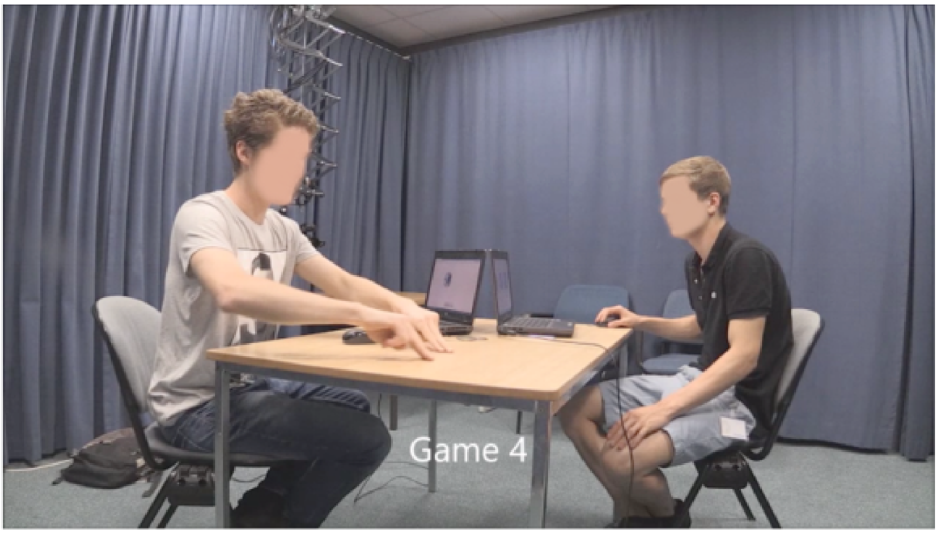

Fig.1: M2
