# Supplementary material for: Multimodality and the origin of a novel communication system in face-to-face interaction
Source: R Soc Open Sci. 2020 Jan 15;7(1):182056. doi: 10.1098/rsos.182056 (PMC7029942; doi:10.1098/rsos.182056)
Supplement: See the attached file for 6 titles [file rsos182056supp1.zip › SupportingMaterials/S4_MainResults_Accuracy.pdf]

Modality effects in a signalling game: Accuracy

Intro

This script uses data compiled by *analyseData.R*.

Load libraries

```
library(lme4)
library(sjPlot)
library(ggplot2)
library(lattice)
library(influence.ME)
library(party)
library(dplyr)
```

Load data

```
d = read.csv("../../data/FinalSignalData.csv")
```

Work out number of turns in each trial.

```
# Number of turns in each trial
numTurns = tapply(d$turnString, d$trialString,
                  function(X){length(unique(X))})
d$numberOfTurns = numTurns[d$trialString]
```

Variable for length of first T1

```
T1L = tapply(d[d$turnType=="T1",]$turnLength,
             d[d$turnType=="T1",]$trialString, head, n=1)
d$T1Length = T1L[d$trialString]
d$T1Length[is.na(d$T1Length)] = mean(d$T1Length,na.rm=T)
d$T1Length.log = log(d$T1Length)
d$T1Length.log = d$T1Length.log - mean(d$T1Length.log)
```

Did matcher respond?

```
matcherResponds = tapply(d$turnType, d$trialString, function(X){
  any(X %in% c("T2","T4","T6","T8",'T10'))
})
d$matcherResponds = matcherResponds[d$trialString]
```

We don't need info on every signal in each turn, just the trial time. Keep only 1st signal in each trial.

```
d = d[!duplicated(d$trialString),]
```

Descriptive stats

Here is a graph showing the distribution of accuracy by conditions:

```
summary = d %>%
  group_by(condition, modalityCondition,game) %>%
  summarise(Accuracy=mean(correct),
            sd=sd(correct),
            ci.w =           qnorm(0.95)*sd/sqrt(length(correct)),
            upper=Accuracy+ci.w,
            lower = Accuracy-ci.w)
```

```
## Warning: package 'bindrcpp' was built under R version 3.3.2
```

```
summary$game = summary$game +1

summary$modalityCondition =
  factor(summary$modalityCondition,
         levels = c("visual",'multi','vocal'),
         labels=c("Gestural","Multimodal","Vocal"))

#ggplot(d, aes(x=trialTotal, y=as.numeric(correct),colour=modalityCondition)) +
#  geom_smooth() + facet_grid(.~condition)

#ggplot(d, aes(x=trialTotal, y=as.numeric(correct),colour=condition)) +
#  geom_smooth() + #facet_grid(.~modalityCondition)

ggplot(summary, aes(x=game, y=Accuracy, group=condition, colour=modalityCondition)) +
  geom_point() +
  geom_errorbar(aes(ymin=lower, ymax=upper)) +
  facet_grid(. ~ condition) +
  stat_summary(fun.y="mean", geom="line", aes(group=modalityCondition))
```

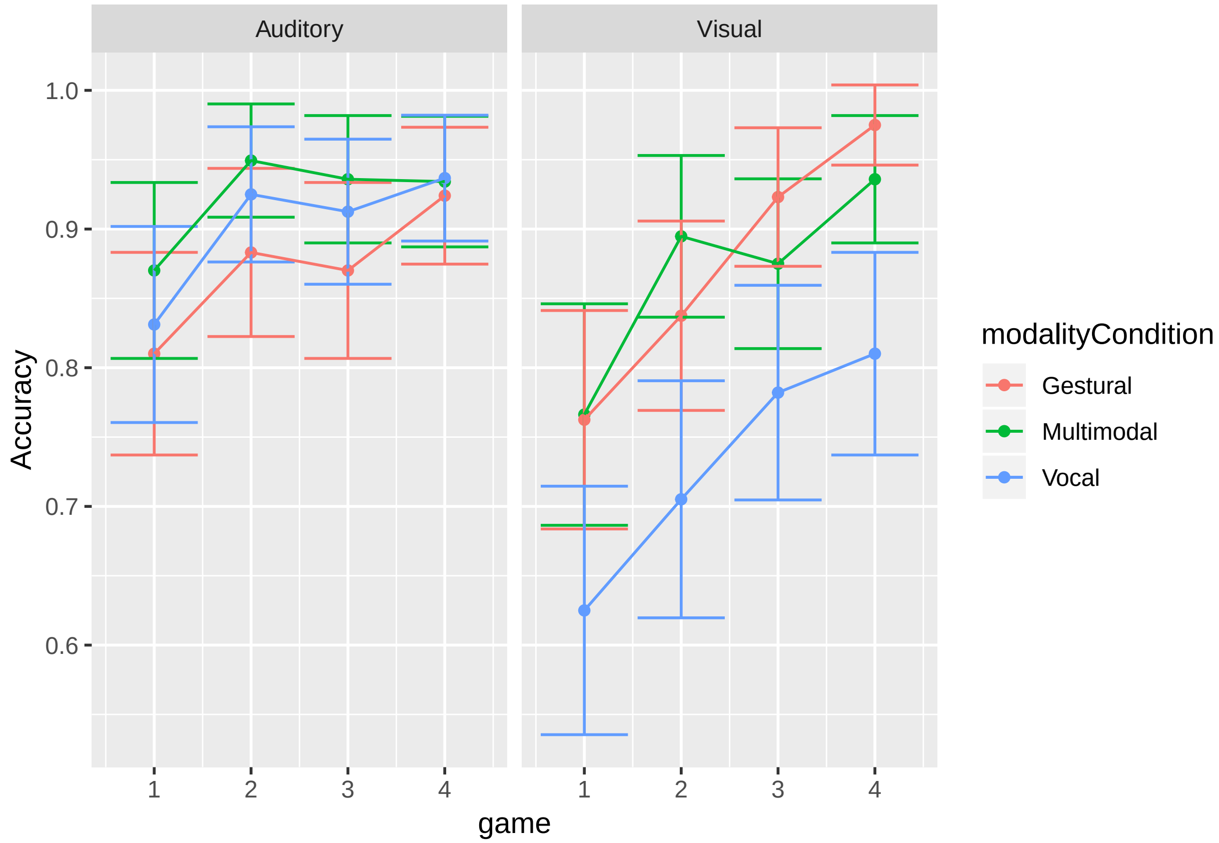


```
gx = ggplot(summary, aes(x=game, y=Accuracy, group=condition, colour=condition, shape=condition)) +
  geom_point() +
  geom_errorbar(aes(ymin=lower, ymax=upper)) +
  facet_grid(. ~ modalityCondition) +
  stat_summary(fun.y="mean", geom="line", aes(group=condition)) +
  scale_colour_brewer(palette="Dark2",name="Stimuli") +
  scale_shape_discrete(name="Stimuli") +
  xlab("Game")
gx
```

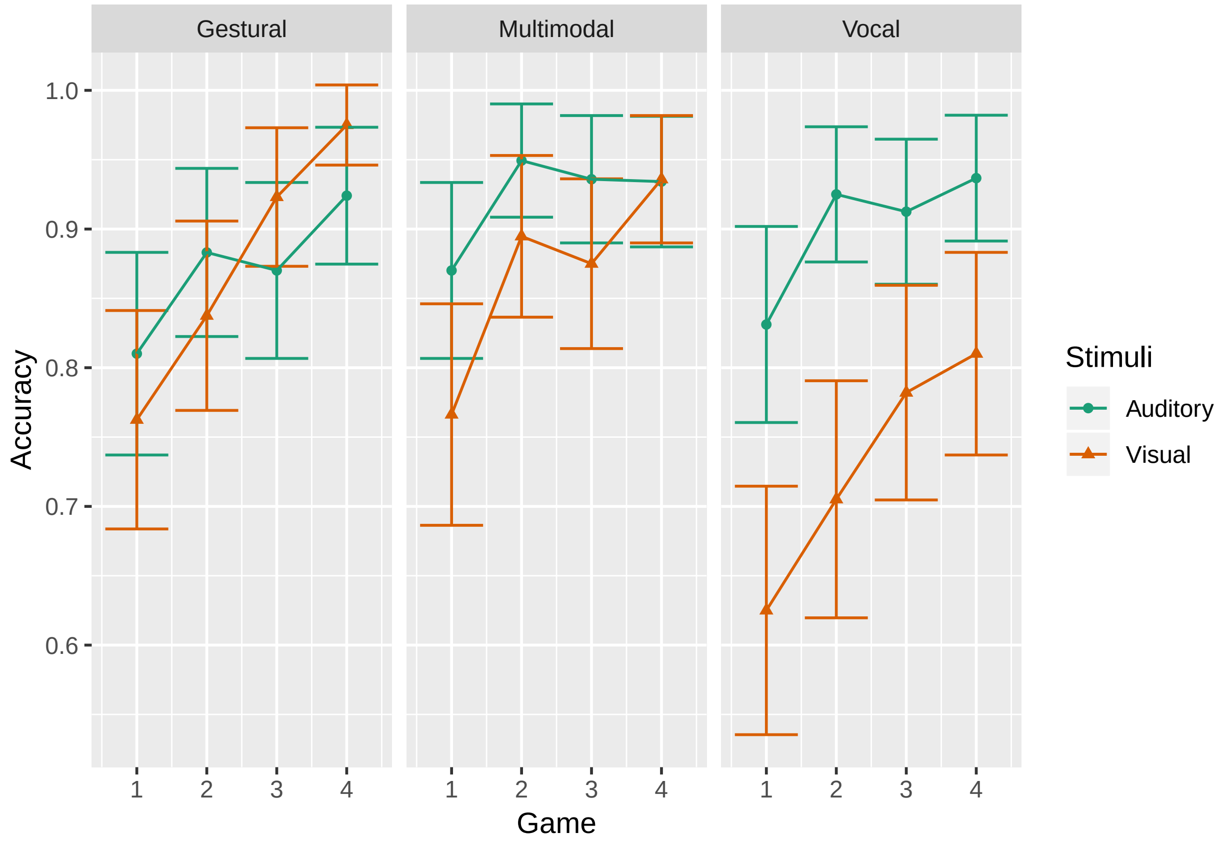


```r
pdf("../../results/graphs/Accuracy_gg.pdf",
    width = 5, height=3)
gx
dev.off()
```

```
## pdf
##   2
```

```r
pd = position_dodge(width=0.5)
gx1 = ggplot(summary, aes(x=game, y=Accuracy, group=condition, colour=modalityCondition)) +
  geom_errorbar(aes(ymin=lower, ymax=upper,group=modalityCondition), width=0.5,position = pd) +
  stat_summary(fun.y="mean", geom="line", aes(group=modalityCondition),position = pd) +
  geom_point(aes(group=modalityCondition,shape=modalityCondition),position=pd) +
  scale_colour_brewer(palette="Set2", name="Condition") +
  scale_shape(name="Condition") +
  ggtitle("Accuracy") +
  theme(panel.grid.major.x = element_blank()) +
  facet_grid(. ~ condition) +
  xlab("Game")

gx1
```

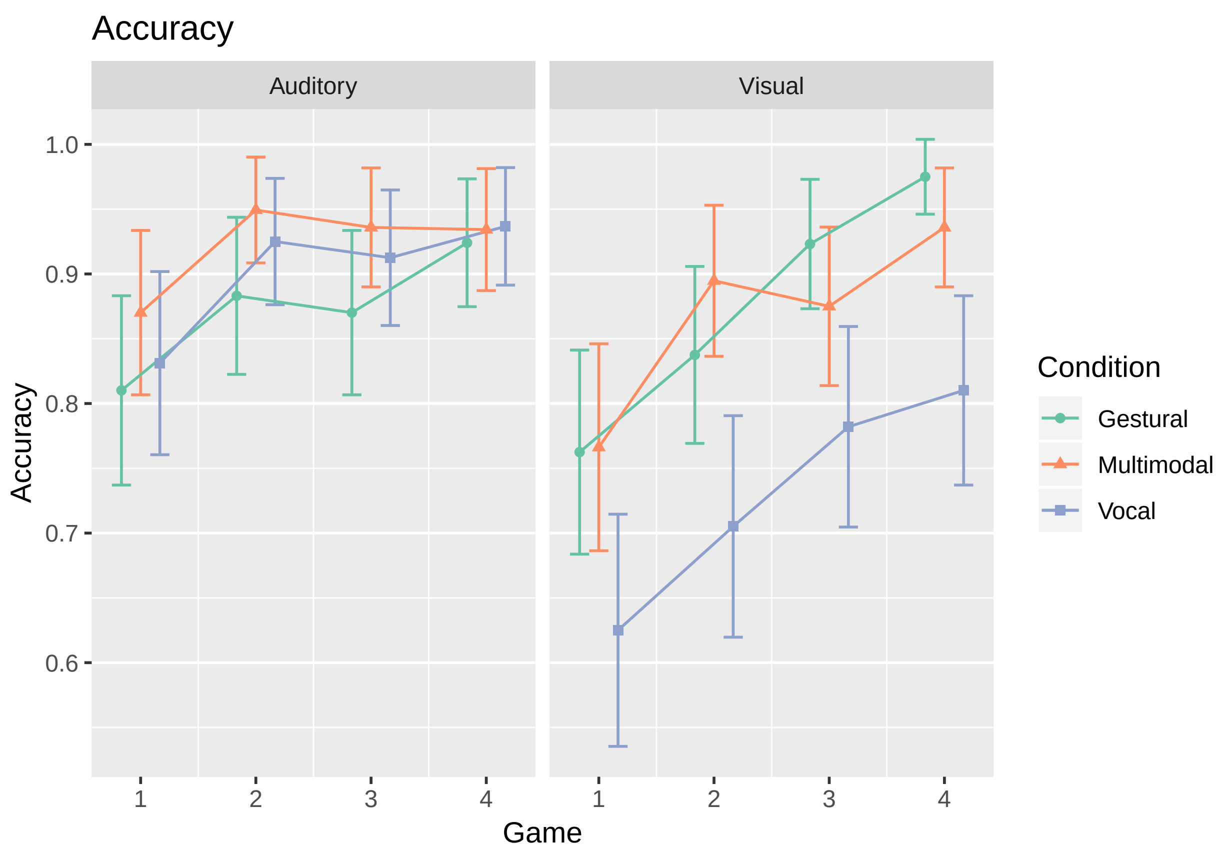


```
pdf("../../results/graphs/Accuracy_gg_alt.pdf",
    width = 5, height=3)
gx1
dev.off()
```

```
## pdf
##   2
```

Make a variable to represent proportion of games played:

```
# Make a variable that represents the number of trials played
d$trialTotal = d$trial + (d$game * (max(d$trial)+1))
# Convert to proportion of games played, so that estimates reflect change per game.
d$trialTotal = d$trialTotal / 16
# Center the trialTotal variable so intercept reflects after the first game
d$trialTotal = d$trialTotal
```

Average accuracy per dyad:

```
av.acc = tapply(d$correct, d$dyadNumber, function(X){sum(X)/length(X)})
mean(av.acc)
```

```
## [1] 0.8613356
```

Make a variable for which stimuli the players experienced first.

```
firstBlock = tapply(as.character(d$condition),d$dyadNumber,head,n=1)
d$firstBlock = as.factor(firstBlock[match(d$dyadNumber,names(firstBlock))])
```

Variable to indicate whether T1 is multimodal.

```
turnD = read.csv("../../data/Final_Turn_data.csv")
turnD = turnD[turnD$turnType=="T1",]
turnD = turnD[turnD$role == "Director",]
d$multimodal = turnD[match(d$trialString, turnD$trialString),]$turnModalityType == "multi"
d$multimodal[is.na(d$multimodal)] = F
```

Make a variable to represent proportion of games played:

```
# Make a variable that represents the number of trials played
d$trialTotal = d$trial + (d$game * (max(d$trial)+1))
# Convert to proportion of games played, so that estimates reflect change per game.
d$trialTotal = d$trialTotal / 16
# Center the trialTotal variable so intercept reflects after the first game
d$trialTotal = d$trialTotal - 2
```

Transformed trial time.

```
d$trialLength.log = log(d$trialLength)
meanLogTrialLength = mean(d$trialLength.log)
d$trialLength.log = d$trialLength.log - meanLogTrialLength
```

Get an idea of the structure of the data from a binary tree:

```
cx = ctree(correct ~ modalityCondition + condition +
            trialTotal +
            trialLength +
            matcherResponds +
            matcherResponds +
            T1Length +
            multimodal+
            firstBlock,
          data=d)
```

```
plot(cx, terminal_panel=node_barplot(cx))
```

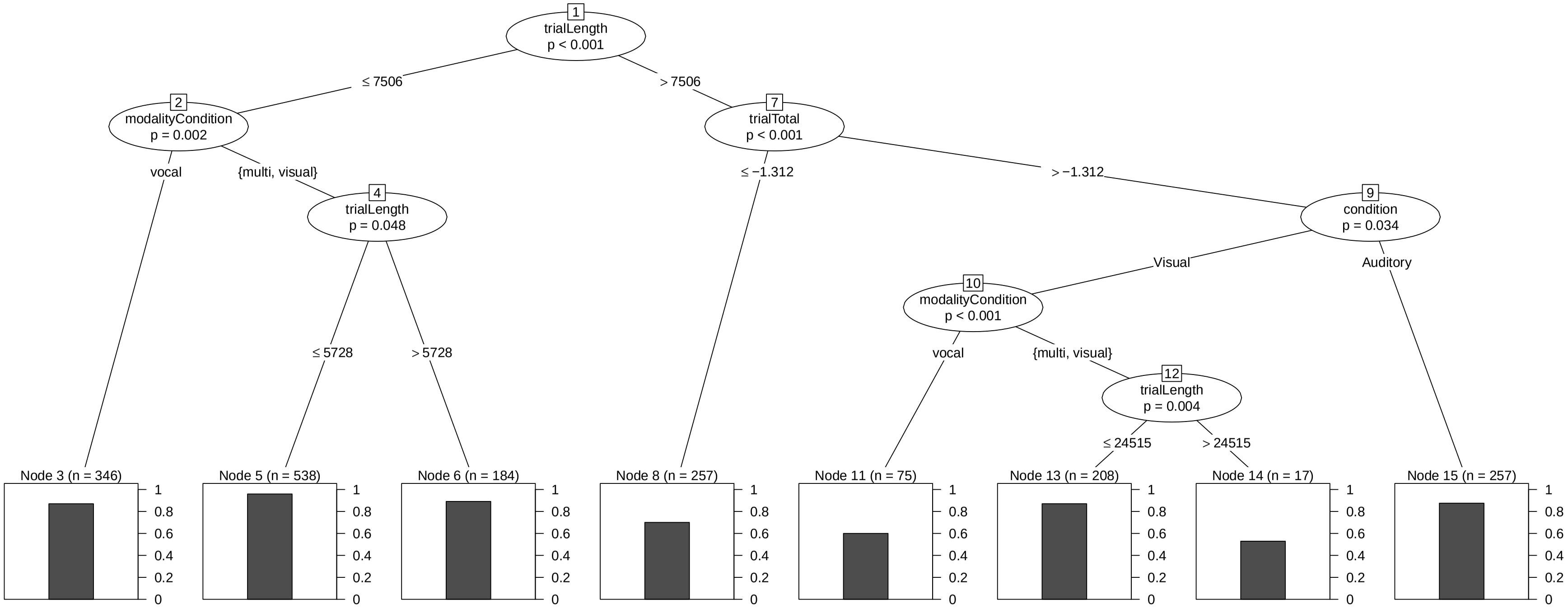

Mixed models

There are celing effects in the data, which reduces variance and makes model convergence difficult. Experimentation revealed that random effects other than random intercepts for dyad and item lead to non-convergence.

The final models do not converge within standard tolerances, but the convergence is acceptable.

```
# No fixed effects

gc = glmerControl(optimizer = "bobyqa" ,optCtrl = list(maxfun=50000))

m0 =  glmer(correct ~ 1 +
            (1 |dyadNumber) +
            (1 |itemId) ,
          data=d, family=binomial,
          control = gc)

game = glmer(correct ~ 1 +
            trialTotal +
            (1 |dyadNumber) +
            (1 |itemId) ,
          data=d, family=binomial,
          control = gc)

trialL = glmer(correct ~ 1 +
            trialTotal +
            trialLength.log+
            (1 |dyadNumber) +
            (1 |itemId) ,
          data=d, family=binomial,
          control = gc)

t1L = glmer(correct ~ 1 +
            trialTotal +
            trialLength.log +
            T1Length.log +
            (1 |dyadNumber) +
            (1 |itemId) ,
          data=d, family=binomial,
          control = gc)

multi = glmer(correct ~ 1 +
            trialTotal +
            trialLength.log +
            T1Length.log +
            multimodal+
            (1 |dyadNumber) +
            (1 |itemId) ,
          data=d, family=binomial,
          control = gc)


mtchTrn = glmer(correct ~ 1 +
            trialTotal +
            trialLength.log +
```

```
            T1Length.log +
            multimodal+
            matcherResponds +
            (1 |dyadNumber) +
            (1 |itemId) ,
          data=d, family=binomial,
          control = gc)

tMtchTr = glmer(correct ~ 1 +
            trialTotal +
            trialLength.log +
            T1Length.log +
            multimodal+
            matcherResponds +
            matcherResponds.cumulative +
            (1 |dyadNumber) +
            (1 |itemId) ,
          data=d, family=binomial,
          control = gc)

con = glmer(correct ~ 1 + condition +
            trialTotal +
            trialLength.log +
            T1Length.log +
            multimodal+
            matcherResponds +
            matcherResponds.cumulative +
            (1 |dyadNumber) +
            (1 |itemId) ,
          data=d, family=binomial,
          control = gc)

mod = glmer(correct ~ 1 + modalityCondition + condition +
            trialTotal +
            trialLength.log +
            T1Length.log +
            multimodal+
            matcherResponds +
            matcherResponds.cumulative +
            (1 |dyadNumber) +
            (1 |itemId) ,
          data=d, family=binomial,
          control = gc)

modXcon = glmer(correct ~ 1 + modalityCondition * condition +
            trialTotal +
            trialLength.log +
            T1Length.log +
            multimodal+
            matcherResponds +
            matcherResponds.cumulative +
            (1 |dyadNumber) +
            (1 |itemId) ,
```

```
          data=d, family=binomial,
          control = gc)

trialLXmo = glmer(correct ~ 1 + modalityCondition * condition +
            trialTotal +
            trialLength.log * modalityCondition+
            T1Length.log +
            multimodal+
            matcherResponds +
            matcherResponds.cumulative +
            (1 |dyadNumber) +
            (1 |itemId) ,
          data=d, family=binomial,
          control = gc)

t1LXmo = glmer(correct ~ 1 + modalityCondition * condition +
            trialTotal +
            trialLength.log * modalityCondition+
            T1Length.log *modalityCondition +
            multimodal+
            matcherResponds +
            matcherResponds.cumulative +
            (1 |dyadNumber) +
            (1 |itemId) ,
          data=d, family=binomial,
          control = gc)

tMaTXmo = glmer(correct ~ 1 + modalityCondition * condition +
            trialTotal +
            trialLength.log * modalityCondition+
            T1Length.log *modalityCondition +
            multimodal+
            matcherResponds +
            matcherResponds.cumulative +
              matcherResponds.cumulative:modalityCondition +
            (1 |dyadNumber) +
            (1 |itemId) ,
          data=d, family=binomial,
          control = gc)


block = glmer(correct ~ 1 + modalityCondition * condition +
            trialTotal +
            trialLength.log * modalityCondition+
            T1Length.log *modalityCondition +
            multimodal+
            matcherResponds +
            matcherResponds.cumulative +
              matcherResponds.cumulative:modalityCondition +
            matcherResponds +
            firstBlock +
            (1 |dyadNumber) +
            (1 |itemId) ,
```

```
             data=d, family=binomial,
             control = gc)
```

Results

Compare the fit of the models:

```
modelComparison = anova(m0,con,mod,modXcon,
                        game, trialL,trialLXmo,
                        t1L, t1LXmo, mtchTrn, tMtchTr,tMaTXmo,
                        multi, block)
x = capture.output(modelComparison)
x[!grepl(": ",x)]
```

```
##  [1] "Models:"
##  [2] "          Df    AIC    BIC  logLik deviance   Chisq Chi Df Pr(>Chisq)    "
##  [3] "m0         3 1405.1 1421.8 -699.56   1399.1                             "
##  [4] "game       4 1355.9 1378.0 -673.95   1347.9 51.2377      1  8.183e-13 ***"
##  [5] "trialL     5 1327.1 1354.8 -658.54   1317.1 30.8054      1  2.852e-08 ***"
##  [6] "t1L        6 1329.0 1362.2 -658.48   1317.0  0.1207      1   0.728314    "
##  [7] "multi      7 1331.0 1369.8 -658.48   1317.0  0.0004      1   0.984192    "
##  [8] "mtchTrn    8 1332.3 1376.7 -658.17   1316.3  0.6269      1   0.428492    "
##  [9] "tMtchTr    9 1327.0 1376.9 -654.51   1309.0  7.3170      1   0.006831 ** "
## [10] "con       10 1326.4 1381.8 -653.21   1306.4  2.6027      1   0.106680    "
## [11] "mod       12 1328.5 1395.0 -652.25   1304.5  1.9097      2   0.384877    "
## [12] "modXcon   14 1306.2 1383.8 -639.10   1278.2 26.2991      2  1.946e-06 ***"
## [13] "trialLXmo 16 1305.6 1394.3 -636.82   1273.6  4.5670      2   0.101926    "
## [14] "t1LXmo    18 1309.1 1408.8 -636.56   1273.1  0.5233      2   0.769786    "
## [15] "tMaTXmo   20 1311.5 1422.3 -635.76   1271.5  1.5940      2   0.450687    "
## [16] "block     21 1313.4 1429.8 -635.71   1271.4  0.1122      1   0.737617    "
## [17] "---"
```

Pick final model for estimates with only significant variables:

```
finalModel = glmer(correct ~ 1 +
            modalityCondition * condition +
            trialTotal +
            trialLength.log +
            matcherResponds.cumulative +
            (1 |dyadNumber) +
            (1 |itemId) ,
          data=d, family=binomial,
          control = gc)
```

Model estimates:

```
summary(finalModel)
```

```
## Generalized linear mixed model fit by maximum likelihood (Laplace
##   Approximation) [glmerMod]
##  Family: binomial  ( logit )
## Formula:
## correct ~ 1 + modalityCondition * condition + trialTotal + trialLength.log +
##     matcherResponds.cumulative + (1 | dyadNumber) + (1 | itemId)
##    Data: d
## Control: gc
##
##      AIC      BIC   logLik deviance df.resid
##   1301.1   1362.0   -639.5   1279.1     1871
```

```
##
## Scaled residuals:
##     Min      1Q  Median      3Q     Max
## -9.2370  0.1303  0.2504  0.4070  2.0033
##
## Random effects:
##  Groups     Name        Variance Std.Dev.
##  itemId     (Intercept) 0.8415   0.9173
##  dyadNumber (Intercept) 0.1465   0.3828
## Number of obs: 1882, groups:  itemId, 16; dyadNumber, 15
##
## Fixed effects:
##                                        Estimate Std. Error z value
## (Intercept)                             2.61527    0.45376   5.764
## modalityConditionvisual                -0.37924    0.38277  -0.991
## modalityConditionvocal                  0.25425    0.39818   0.639
## conditionVisual                        -0.55322    0.55680  -0.994
## trialTotal                              0.21954    0.07547   2.909
## trialLength.log                        -0.96227    0.14685  -6.553
## matcherResponds.cumulative              0.08288    0.04338   1.910
## modalityConditionvisual:conditionVisual  0.66413    0.39358   1.687
## modalityConditionvocal:conditionVisual  -1.11930    0.39222  -2.854
##                                        Pr(>|z|)
## (Intercept)                            8.24e-09 ***
## modalityConditionvisual                 0.32180
## modalityConditionvocal                  0.52313
## conditionVisual                         0.32043
## trialTotal                              0.00363 **
## trialLength.log                        5.65e-11 ***
## matcherResponds.cumulative              0.05607 .
## modalityConditionvisual:conditionVisual  0.09152 .
## modalityConditionvocal:conditionVisual   0.00432 **
## ---
## Signif. codes:  0 '***' 0.001 '**' 0.01 '*' 0.05 '.' 0.1 ' ' 1
##
## Correlation of Fixed Effects:
##               (Intr) mdltyCndtnvs mdltyCndtnvc cndtnV trlTtl trlLn.
## mdltyCndtnvs   -0.442
## mdltyCndtnvc   -0.490  0.510
## conditinVsl    -0.626  0.261        0.211
## trialTotal      0.163 -0.008       -0.141        0.010
## trlLngth.lg     0.050 -0.105       -0.143       -0.069  0.385
## mtchrRspnd.    -0.211 -0.123        0.214       -0.114 -0.384 -0.117
## mdltyCndtnvs:V  0.245 -0.581       -0.281       -0.409 -0.045  0.043
## mdltyCndtnvc:V  0.246 -0.379       -0.562       -0.418 -0.008  0.204
##               mtchR. mdltyCndtnvs:V
## mdltyCndtnvs
## mdltyCndtnvc
## conditinVsl
## trialTotal
## trlLngth.lg
## mtchrRspnd.
## mdltyCndtnvs:V  0.204
## mdltyCndtnvc:V  0.173  0.583
```

```r
# number of correctly categorised trials
sum((predict(finalModel)>0) == d$correct)/nrow(d)
```

```
## [1] 0.8666312
```

Plot the fixed effects

Relabel the effects:

```r
feLabels = matrix(c(
"(Intercept)"              ,"Intercept"      , NA,
"modalityConditionvisual" ,"Visual modality", "mod",
"modalityConditionvocal"  , "Acoustic modality", "mod",
"conditionVisual" , "Visual stimuli","con",
"trialTotal"             , "Game","game",
"modalityConditionvisual:conditionVisual"  , "Visual modality:Visual stimuli", "modXcon",
"modalityConditionvocal:conditionVisual" , "Acoustic modality:Visual stimuli","modXcon",
"firstBlockVisual","Visual stims first","block",
"trialLength.log","Trial length","trialL",
"modalityConditionvisual:trialLength.log","Visual modality:Trial length",'trialLXmo',
"modalityConditionvocal:trialLength.log","Acoustic modality:Trial length",'trialLXmo',
"multimodalTRUE","Multimodal T1","multi",
"trialLength.log",'Trial Length','trialL',
"T1Length.log","T1 length","t1L",
"modalityConditionvisual:T1Length.log","T1 length:Visual modality","t1LXmo",
"modalityConditionvocal:T1Length.log","T1 length:Acoustic modality","t1LXmo",
"matcherRespondsTRUE","Matcher Responds",'mtchTrn',
"matcherResponds.cumulative","Total interaction","tMtchTr",
"modalityConditionvisual:matcherResponds.cumulative","Total interaction:Visual Modality","tMaTXmo",
"modalityConditionvocal:matcherResponds.cumulative","Total interaction:Vocal Modality","tMaTXmo"
), ncol=3, byrow = T)
feLabels1 = as.vector(feLabels[match(names(fixef(finalModel)),feLabels[,1]),1])
feLabels2 = as.vector(feLabels[match(names(fixef(finalModel)),feLabels[,1]),2])
feModel = as.vector(feLabels[match(names(fixef(finalModel)),feLabels[,1]),3])

sig = modelComparison$`Pr(>Chisq)`
names(sig) = rownames(modelComparison)

sig.data = data.frame(
  estimate = fixef(finalModel),
  y=1:length(fixef(finalModel)),
  sig=sig[feModel])
sig.data$fade = sig.data$sig > 0.05
```

Plot the strength of the fixed effects:

```r
x = sjp.glmer(finalModel, 'fe',
         show.intercept = T,
         sort.est=NULL,
         axis.labels = feLabels2[2:length(feLabels2)],
         axis.title="Odds of correct selection",
         geom.colors = c(1,1),
         show.values = F,
         show.p = F,
```

```
         fade.ns = T,
         string.interc="Intercept",
         prnt.plot = F)

x$plot.list[[1]]$data$fade = sig.data$fade

x$plot.list[[1]]
```

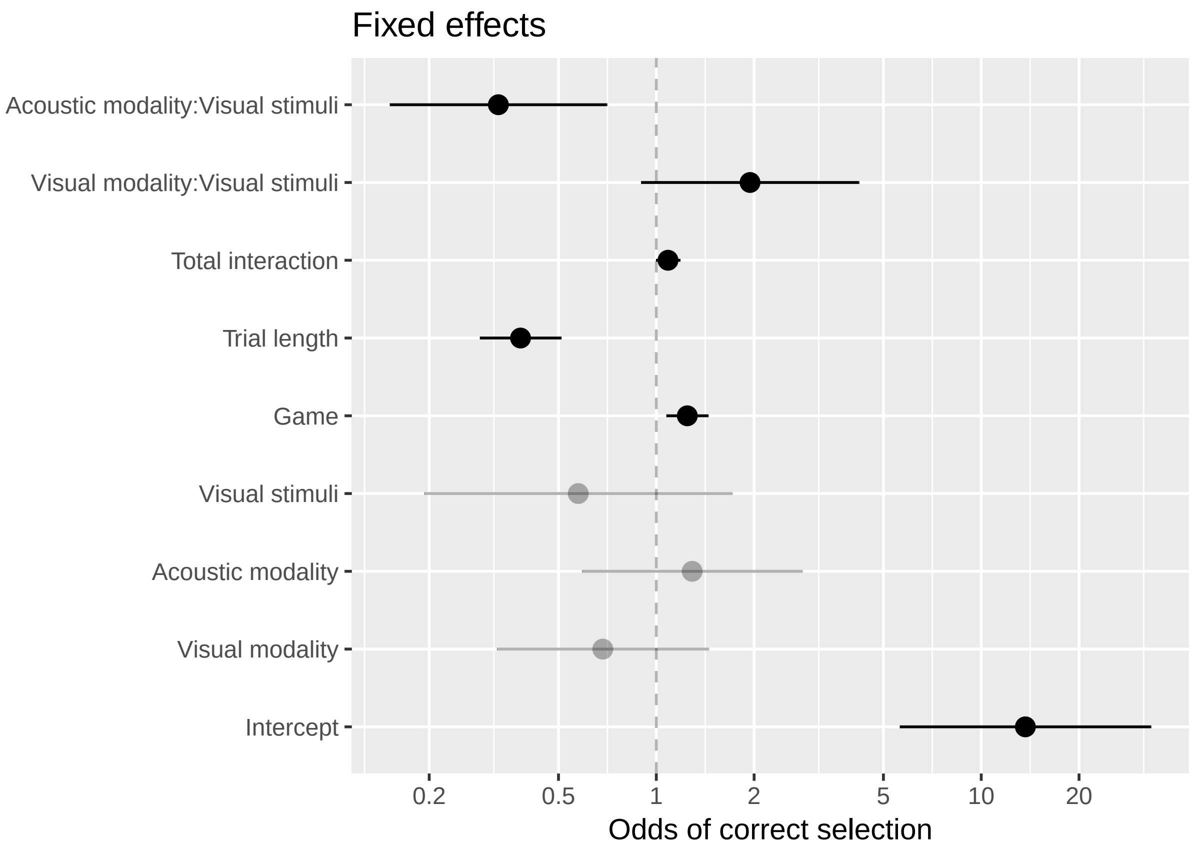

Table of results

```
x = as.data.frame(summary(finalModel)$coef)
mc = as.data.frame(modelComparison)
write.csv(cbind(x,mc[feModel,]), "../../results/tables/Accuracy_FixedEffects.csv")
```

Random effects

```
sjp.glmer(finalModel,'re', sort.est ="sort.all",
          facet.grid = F,
          geom.colors= c(1,1))
```

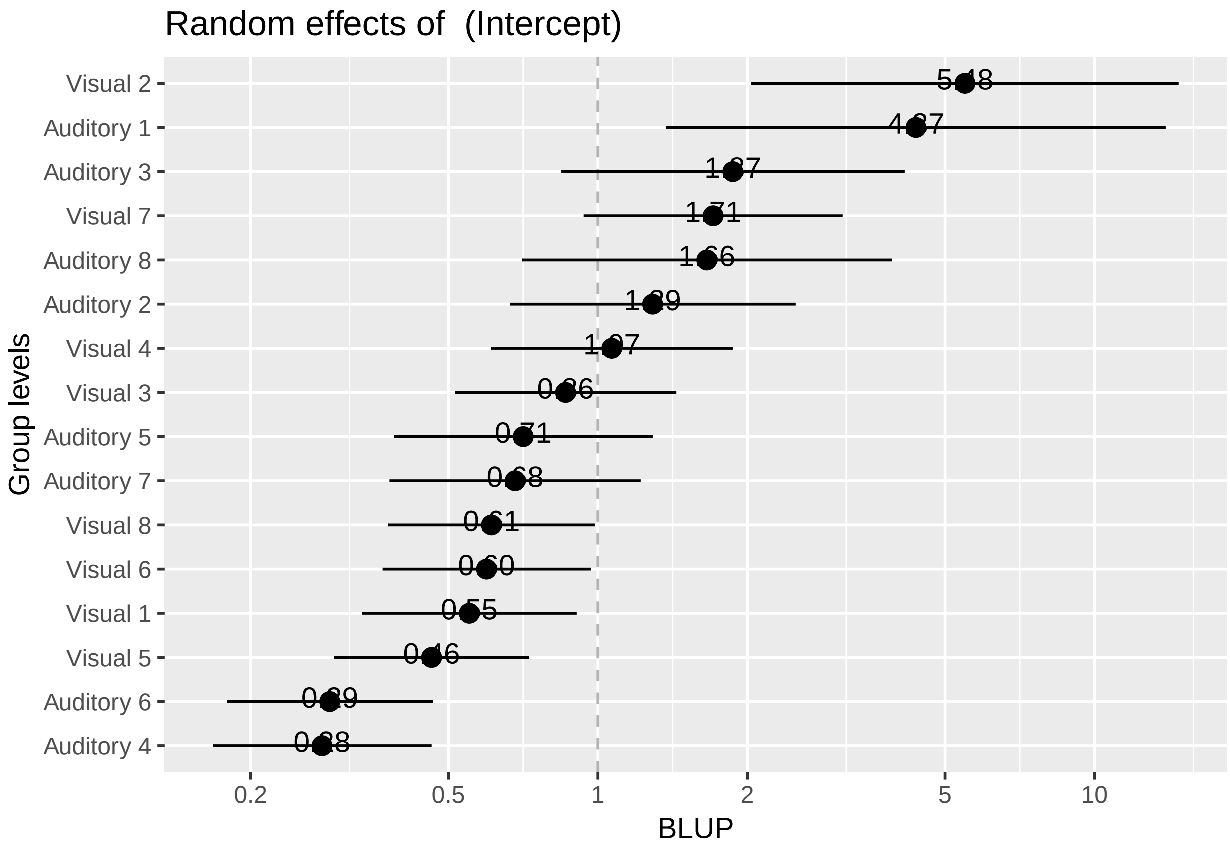

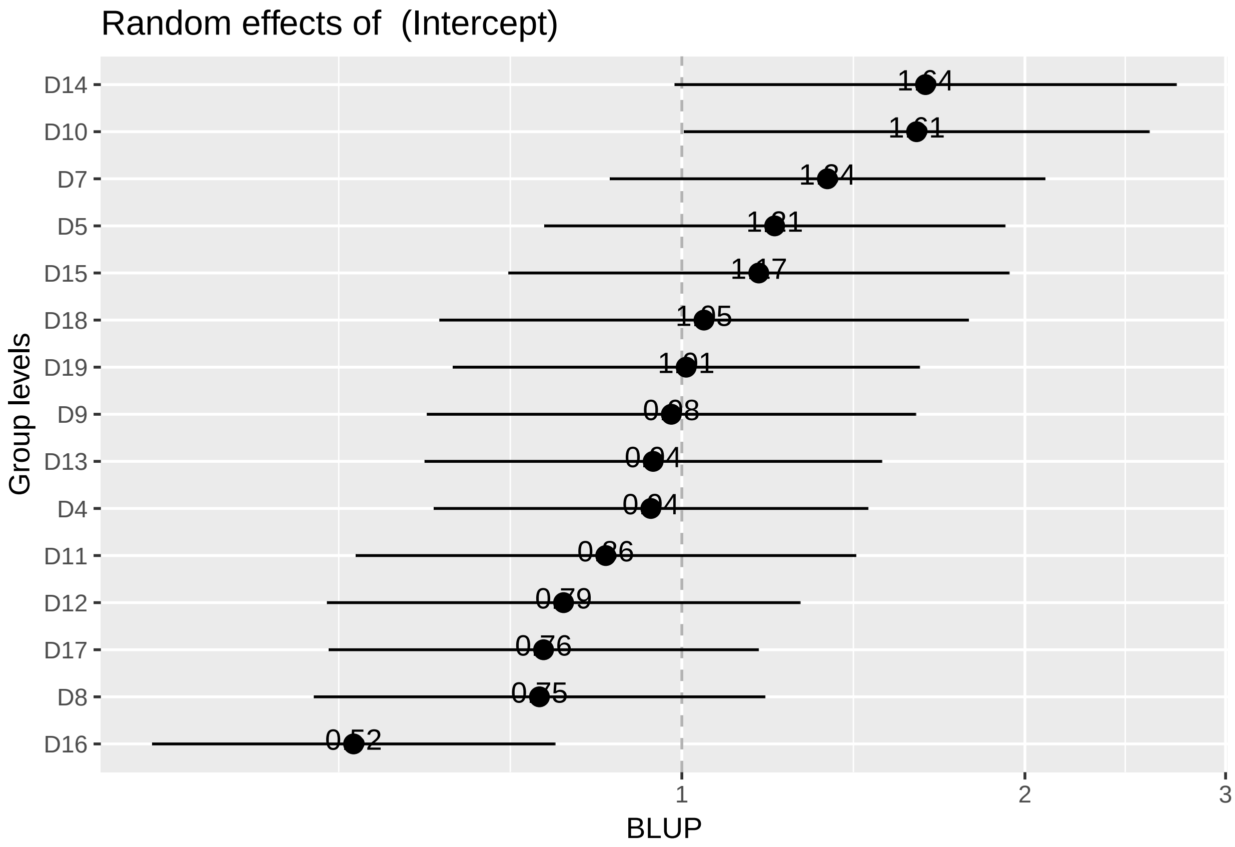

qq-plots of random effects

```r
sjp.glmer(finalModel, type = "re.qq")
```

```
## Testing for normal distribution. Dots should be plotted along the line.
```

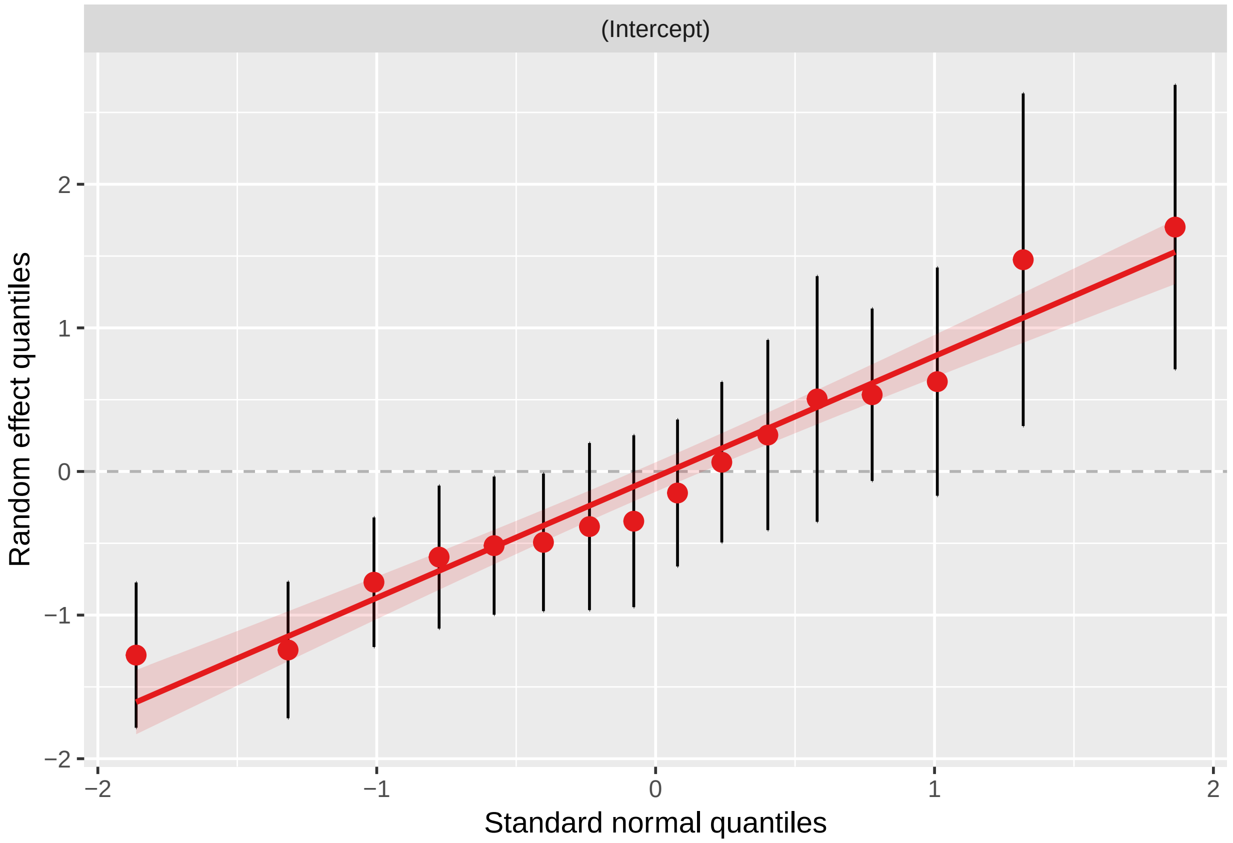
(Intercept)
Random effect quantiles
Standard normal quantiles
2
1
0
−1
−2
−2
−1
0
1
2

Plots

```r
gx = sjp.glmer(finalModel, 'eff', vars=c("matcherResponds.cumulative"),
               title = '', prnt.plot = F,
               show.ci=T, facet.grid = F)

pdf("../../results/graphs/CumulativeMatcherTurns.pdf",
    width=4,height=4)
gx$plot +
  xlab("Number of previous trials where\nmatcher responded") +
  ylab("Probability of correct choice") +
  #scale_x_continuous(breaks = c(0,5,10,15)) +
  coord_cartesian(xlim=c(0,15))
dev.off()
```
