# Supplementary material for: Multimodality and the origin of a novel communication system in face-to-face interaction
Source: R Soc Open Sci. 2020 Jan 15;7(1):182056. doi: 10.1098/rsos.182056 (PMC7029942; doi:10.1098/rsos.182056)
Supplement: See the attached file for 6 titles [file rsos182056supp1.zip › SupportingMaterials/S5_MainResults_Efficiency.pdf]

Modality effects in a signalling game

Intro

The main data used in this analysis comes from `../../data/FinalSignalData.csv` (compiled by `analyseData.R`). Each row represents one signal, but this script only keeps one signal per trial, and the rest of the analysis is on the trial-by-trial level. The variables in the data are as follows (some are calculated in the script below):

- X: ID
- filename: Filename of the ELAN file
- dyadNumber: ID of the participant dyad
- condition: Stimuli type (Auditory or Visual)
- game: Game number (0-3)
- trial: Trial number (0-15)
- target: Target stimuli shown to the director
- choice: Meaning chosen by the matcher
- correct: True if the matcher's choice is correct
- trialStart, trialEnd, trialLength: Start, end and length of trial in milliseconds
- trialValue: A unique string that represents data from the trial. Numbers in the curly brackets represent the choices given to the matcher
- startOfNextTrial: Timestamp for next trial, used in processing the data.
- turnStart, turnEnd, turnLength: the start, end and length of the turn in milliseconds.
- signalStart, signalEnd, signalLength: the start, end and length of the signal.
- signalType: Annotation value in ELAN, not meaningful
- tiralString: Unique string to identify trial
- modalityCondition: The condition for the dyad (multi= multimodal, visual=gesture only, vocal=vocal only)
- playerId: Unique ID for the participant producing the signal
- itemId: Unique ID for the target stimulus
- turnString: Unque ID for the turn
- matcherResponds: Does matcher take a turn in this trial?
- matcherResponds.cumulative: The (scaled) number of previous trials that a has responded.
- T1Length, T1Length.log: Length and log length of the director's first turn.
- trialTotal: Number of trials played so far, scaled (and centered) to represent number of games played.
- firstBlock: Block order
- incorrect: Was the matcher's choice incorrect?
- multimodal: Was the director's first turn multimodal?

Load libraries

```
library(lme4)
library(sjPlot)
library(ggplot2)
library(lattice)
library(influence.ME)
library(dplyr)
```

Load data

```
d = read.csv("../../data/FinalSignalData.csv")
```

Variable for length of first T1

```
T1L = tapply(d[d$turnType=="T1",]$turnLength,
             d[d$turnType=="T1",]$trialString, head, n=1)
d$T1Length = T1L[d$trialString]
d$T1Length[is.na(d$T1Length)] = mean(d$T1Length,na.rm=T)
d$T1Length.log = log(d$T1Length)
d$T1Length.log = d$T1Length.log - mean(d$T1Length.log)
```

We don't need info on every signal in each turn, just the trial time. Keep only 1st signal in each trial.

```
d = d[!duplicated(d$trialString),]
```

Descriptive stats

Make a variable to represent proportion of games played:

```
# Make a variable that represents the number of trials played
d$trialTotal = d$trial + (d$game * (max(d$trial)+1))
# Convert to proportion of games played, so that estimates reflect change per game.
d$trialTotal = d$trialTotal / 16
```

Here is a graph showing the distribution of trial lengths by conditions:

```
summary = d %>%
  group_by(condition, modalityCondition,game) %>%
  summarise(Efficiency=mean(trialLength),
            sd=sd(trialLength),
            ci.w =           qnorm(0.95)*sd/sqrt(length(trialLength)),
            upper=Efficiency+ci.w,
            lower = Efficiency-ci.w)
```

```
## Warning: package 'bindrcpp' was built under R version 3.3.2
```

```
summary$game = summary$game +1

summary$modalityCondition =
  factor(summary$modalityCondition,
         levels = c("visual",'multi','vocal'),
         labels=c("Gestural","Multimodal","Vocal"))

ggplot(d, aes(x=trialTotal, y=trialLength,colour=modalityCondition)) +
  geom_smooth() + facet_grid(.~condition)
```

```
## `geom_smooth()` using method = 'loess' and formula 'y ~ x'
```

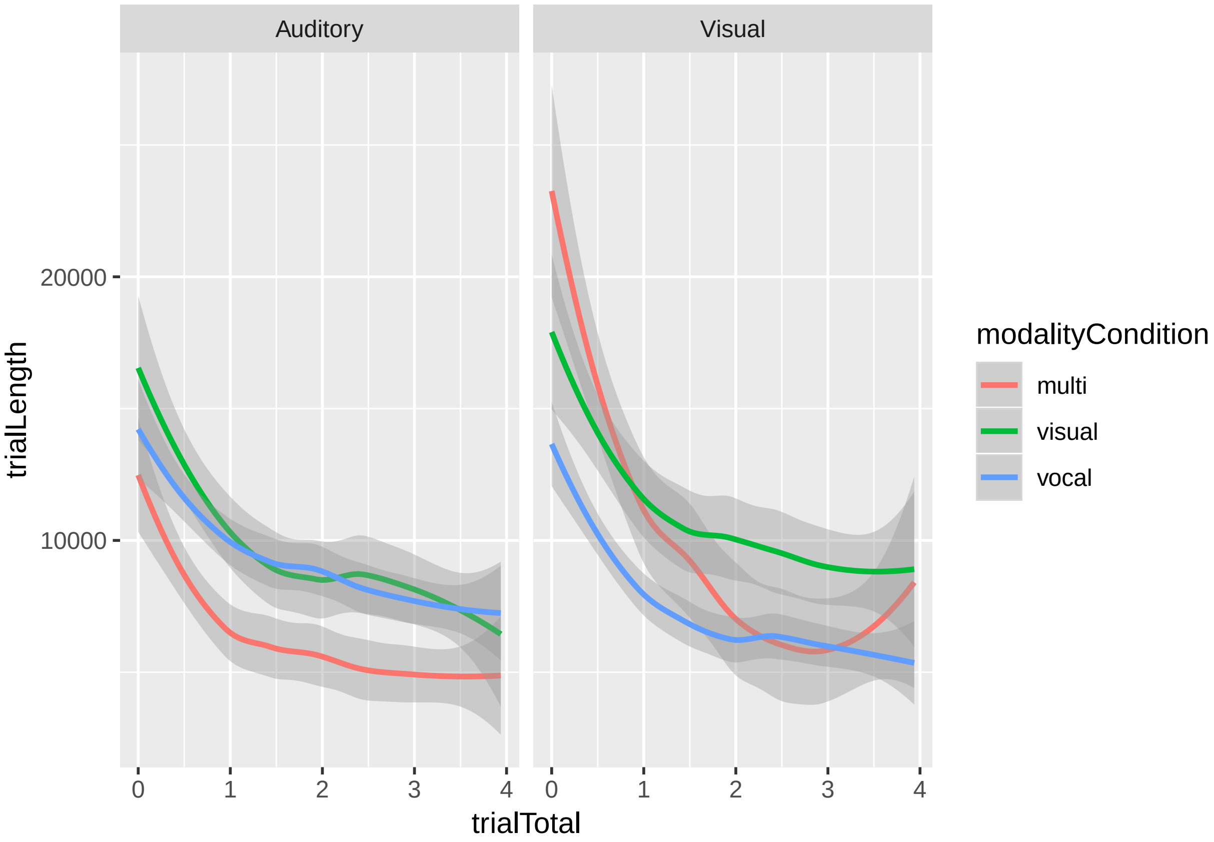


```
ggplot(d, aes(x=trialTotal, y=trialLength,colour=condition)) +
  geom_smooth() + facet_grid(.~modalityCondition)
```

```
## `geom_smooth()` using method = 'loess' and formula 'y ~ x'
```

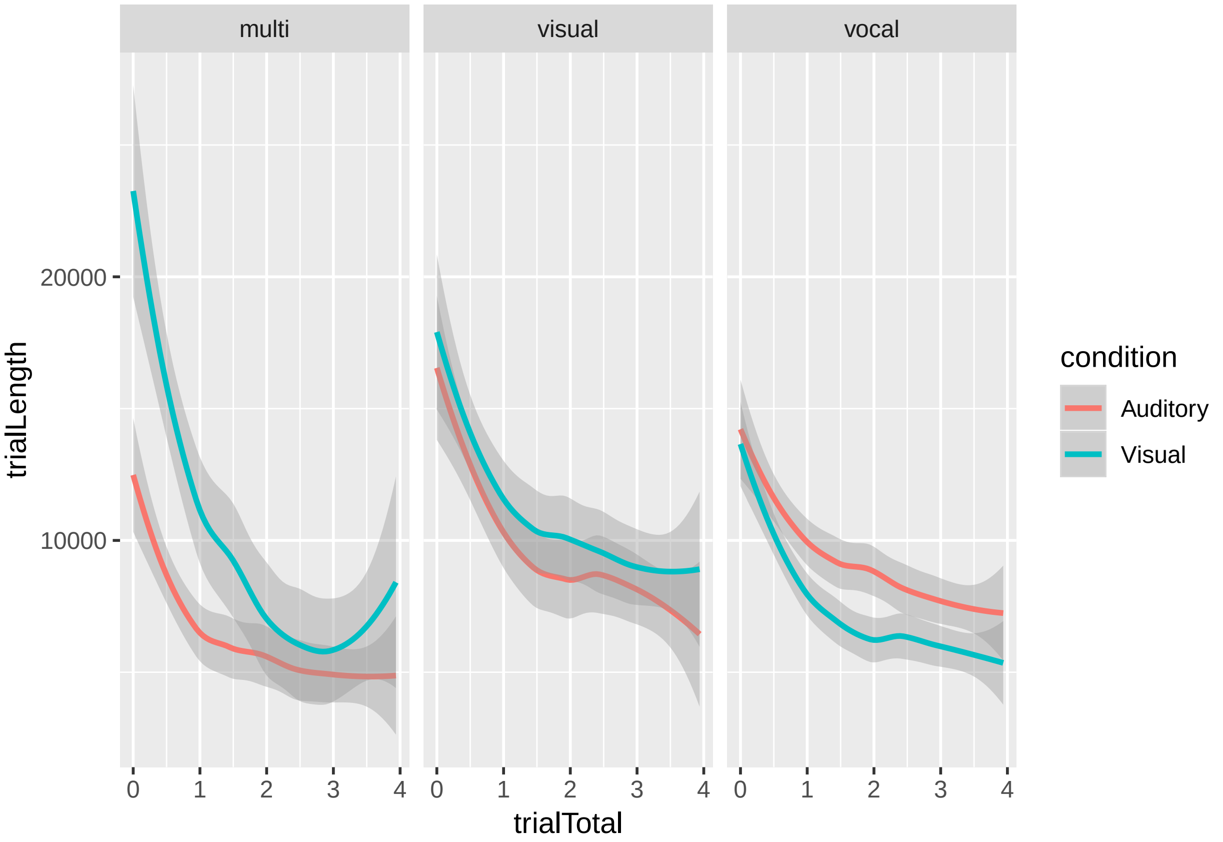


```
pd = position_dodge(width=0.5)
gx1 = ggplot(summary, aes(x=game, y=Efficiency, group=condition, colour=modalityCondition)) +
  geom_errorbar(aes(ymin=lower, ymax=upper,group=modalityCondition), width=0.5,position=pd) +
  facet_grid(. ~ condition) +
  stat_summary(fun.y="mean", geom="line", aes(group=modalityCondition),position=pd) +
    geom_point(aes(group=modalityCondition,shape=modalityCondition),position=pd) +
  scale_colour_brewer(palette="Set2", name="Condition") +
  scale_shape(name="Condition") +
  theme(panel.grid.major.x = element_blank()) +
  ggtitle("Efficiency") +
  xlab("Game") +
  ylab("Trial length (ms)")

gx1
```

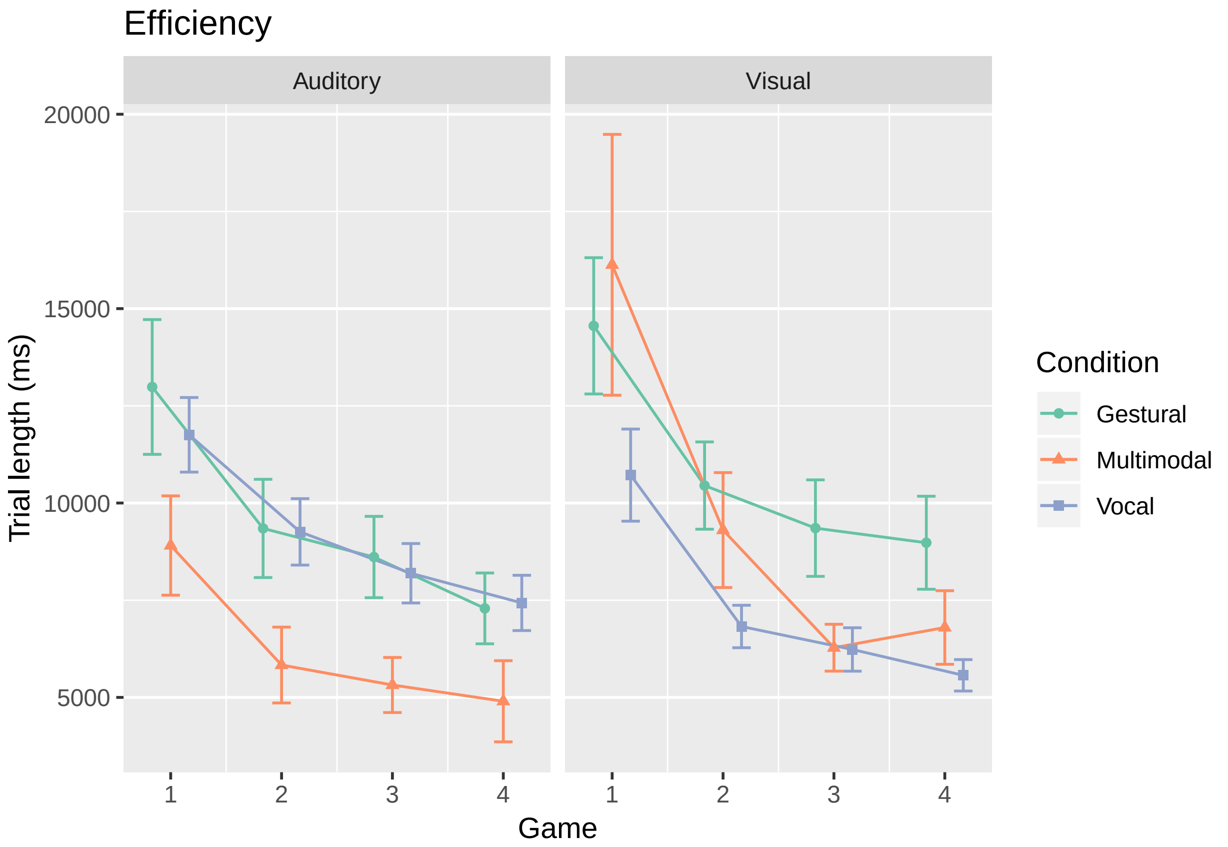


```
pdf("../../results/graphs/Efficiency_gg.pdf",
    width = 5, height=3)
gx1
dev.off()
```

```
## pdf
##   2
```

```
gx2 = ggplot(summary, aes(x=game, y=Efficiency, group=condition, colour=condition, shape=condition)) +
  geom_errorbar(aes(ymin=lower, ymax=upper), width=0.5) +
  facet_grid(. ~ modalityCondition) +
  stat_summary(fun.y="mean", geom="line", aes(group=condition)) +
    geom_point() +
  scale_colour_discrete(name="Stimuli") +
  scale_shape_discrete(name="Stimuli") +
  xlab("Game")

gx2
```

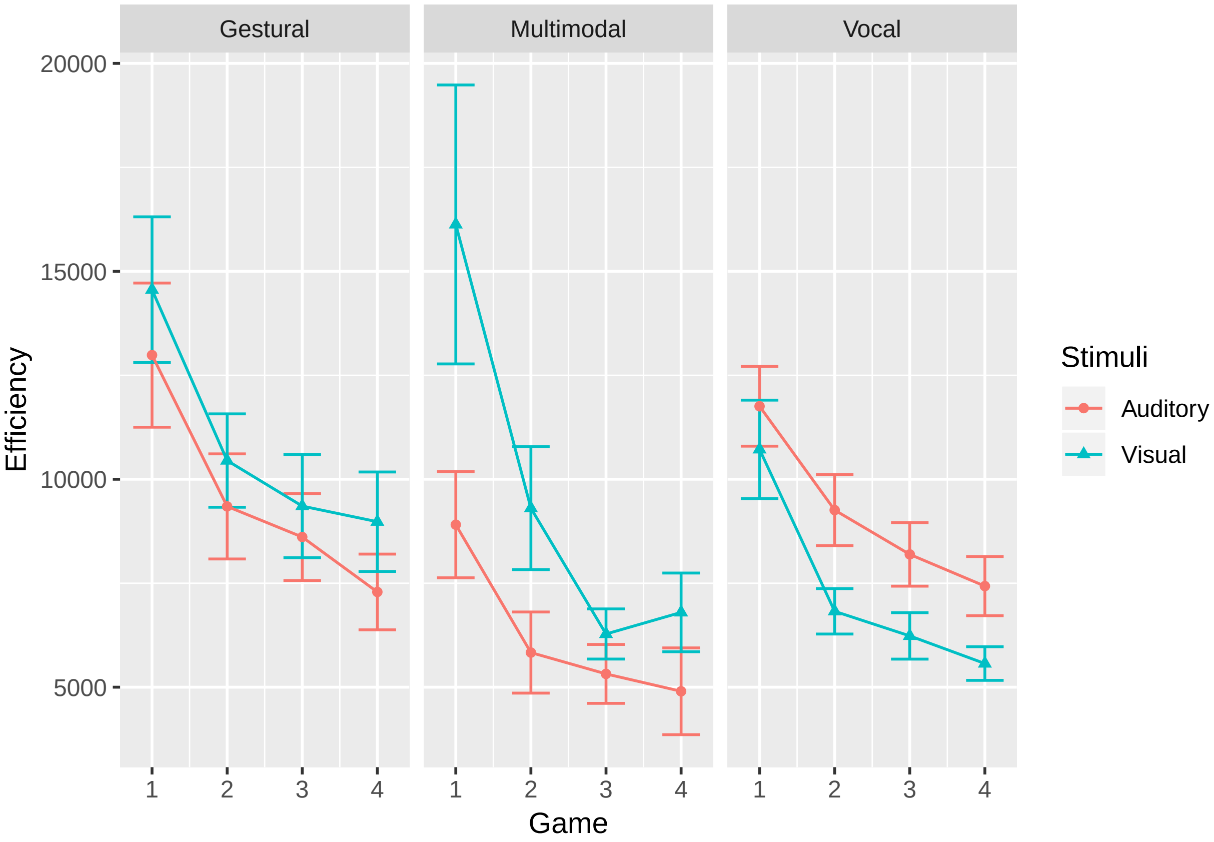


```
pdf("../../results/graphs/Efficiency_gg_alt.pdf",
    width = 5, height=3)
gx2
dev.off()
```

```
## pdf
##   2
```

Average trial time for the whole experiment:

```
mean(d$trialLength)
```

```
## [1] 8795.327
```

```
sd(d$trialLength)
```

```
## [1] 7239.617
```

The distribution of trial times is very skewed:

```
hist(d$trialLength)
```

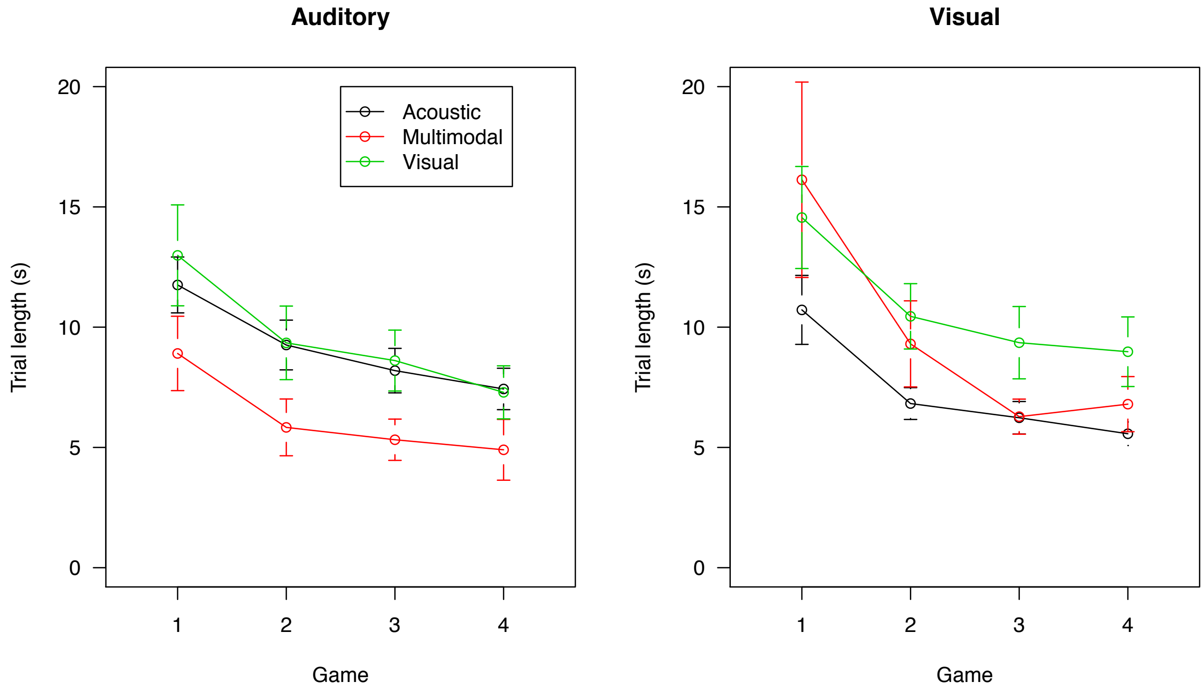

Figure 1: The efficiency of trials in different conditions

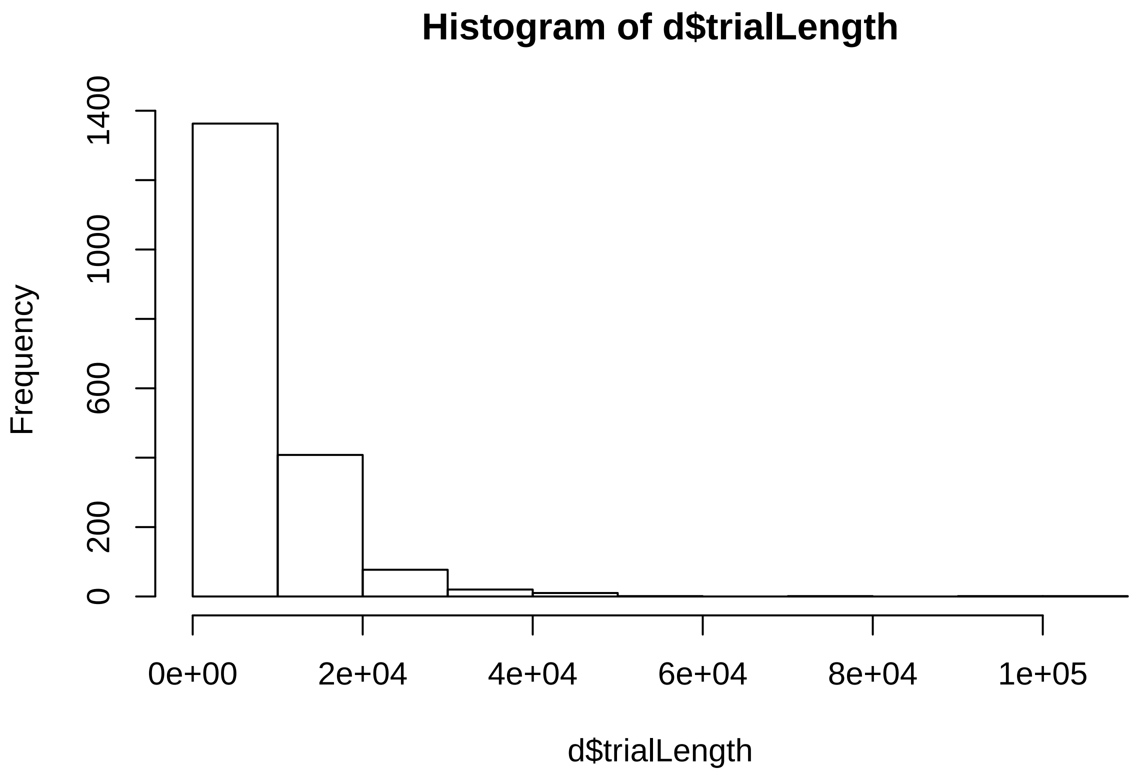

So we transform it using a log transform, then center the data.

```
d$trialLength.log = log(d$trialLength)
meanLogTrialLength = mean(d$trialLength.log)
d$trialLength.log = d$trialLength.log - meanLogTrialLength
hist(d$trialLength.log)
```

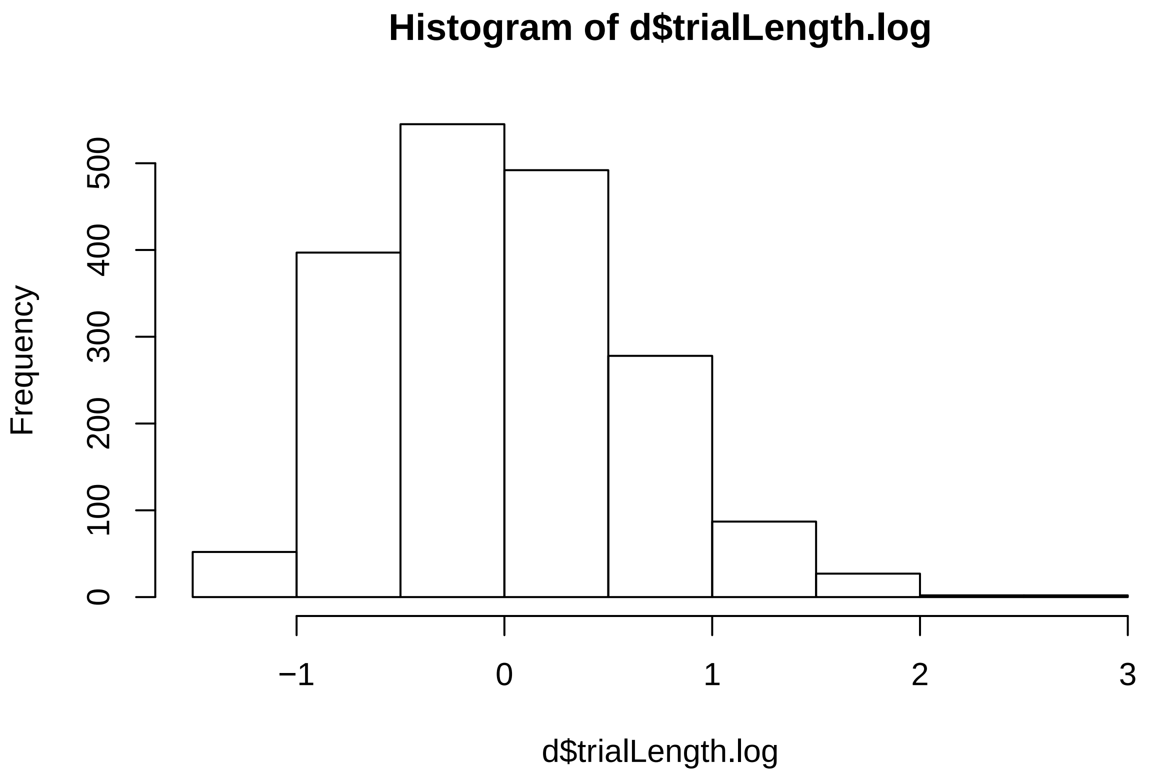


```
# Center the trialTotal variable so intercept reflects after the first game
d$trialTotal = d$trialTotal - 2

matcherResponds.cumulative.mean = mean(d$matcherResponds.cumulative)

d$matcherResponds.cumulative = d$matcherResponds.cumulative - matcherResponds.cumulative.mean

d$matcherResponds = factor(d$matcherResponds)
```

Make a variable for which stimuli the players experienced first.

```
firstBlock = tapply(as.character(d$condition),d$dyadNumber,head,n=1)
d$firstBlock = as.factor(firstBlock[match(d$dyadNumber,names(firstBlock))])
```

Reorder some levels so that the intercept reflects the most frequent condition.

```
d$incorrect = !d$correct
```

Variable for whether T1 was a multimodal signal.

```
turnD = read.csv("../../data/Final_Turn_data.csv")
turnD = turnD[turnD$turnType=="T1",]
turnD = turnD[turnD$role == "Director",]
d$multimodal = turnD[match(d$trialString, turnD$trialString),]$turnModalityType == "multi"
d$multimodal[is.na(d$multimodal)] = F
```

Mixed models

Make a series of models with random effects for dyad, director (nested within dyad) and item.

Not all random slopes are appropriate. For example, items are used in only one stimulus condition, so a random slope for condition by item is not appropriate. Similarly, each dyad only plays in one modality condition.

It is reasonable to have a random slope for trial by dyad, but this caused unreliable model convergence, so is not included.

The final random slopes were for condition and incorrectness by dyad/player, and modality condition by item.

```
# No fixed effects
m0 =  lmer(trialLength.log ~ 1 +
            (1 + condition + incorrect |dyadNumber/playerId) +
            (1 + modalityCondition|itemId),
          data=d, REML = FALSE)
```

Now we add a series of possible confounding factors such as whether the matcher responds. We add the main experimental factors at the end to ensure that they're really contributing to the model over and above the confounds.

```
# Add number of matcher turns
mtchTrn = lmer(trialLength.log ~ 1 +
                matcherResponds +
            (1 + condition + incorrect |dyadNumber/playerId) +
            (1 + modalityCondition|itemId),
          data=d, REML = FALSE)

tMtchTr = lmer(trialLength.log ~ 1 +
                matcherResponds +
                matcherResponds.cumulative +
            (1 + condition + incorrect |dyadNumber/playerId) +
            (1 + modalityCondition|itemId),
          data=d, REML = FALSE)

# Add whether the response was incorrect
incor = lmer(trialLength.log ~ 1 +
            matcherResponds +
            matcherResponds.cumulative +
            incorrect +
            (1 + condition + incorrect |dyadNumber/playerId) +
            (1 + modalityCondition|itemId),
          data=d, REML = FALSE)
```

```
# Add multimodal signal

multim = lmer(trialLength.log ~ 1 +
            matcherResponds +
            matcherResponds.cumulative +
            incorrect +
            multimodal +
            (1 + condition + incorrect |dyadNumber/playerId) +
            (1 + modalityCondition|itemId),
          data=d, REML = FALSE)
```

```r
# Add effect of trial

game = lmer(trialLength.log ~ 1 +
            trialTotal +
            matcherResponds +
            matcherResponds.cumulative +
            incorrect +
            multimodal +
            (1 + condition + incorrect |dyadNumber/playerId) +
            (1 + modalityCondition|itemId),
          data=d, REML = FALSE)

# Add the quadratic effect of trial
gamQuad = lmer(trialLength.log ~ 1 +
            trialTotal + I(trialTotal^2) +
            matcherResponds +
            matcherResponds.cumulative +
            incorrect +
            multimodal +
            (1 + condition + incorrect |dyadNumber/playerId) +
            (1 + modalityCondition|itemId),
          data=d, REML = FALSE)
```

```r
# Add modality condition
modality =  lmer(trialLength.log ~ 1 + modalityCondition +
            trialTotal + I(trialTotal^2) +
            matcherResponds +
            matcherResponds.cumulative +
            incorrect +
            multimodal +
            (1 + condition + incorrect |dyadNumber/playerId) +
            (1 + modalityCondition|itemId),
          data=d, REML = FALSE)
# Add stimulus condition
cond = lmer(trialLength.log ~ 1 + modalityCondition + condition +
            trialTotal + I(trialTotal^2) +
            matcherResponds +
            matcherResponds.cumulative +
            incorrect +
            multimodal +
            (1 + condition + incorrect |dyadNumber/playerId) +
            (1 + modalityCondition|itemId),
          data=d, REML = FALSE)

# Add interaction between modality and stimulus condition
modXcond = lmer(trialLength.log ~ 1 + modalityCondition*condition +
           trialTotal + I(trialTotal^2) +
            matcherResponds +
            matcherResponds.cumulative +
            incorrect +
            multimodal +
            (1 + condition + incorrect |dyadNumber/playerId) +
            (1 + modalityCondition|itemId),
          data=d, REML = FALSE)
```

```r
# Add interaction between condition and trial
conXgame = lmer(trialLength.log ~ 1 + modalityCondition*condition +
            trialTotal + I(trialTotal^2) +
              condition:trialTotal +
            matcherResponds +
            matcherResponds.cumulative +
            incorrect +
            multimodal +
            (1 + condition + incorrect |dyadNumber/playerId) +
            (1 + modalityCondition|itemId),
          data=d, REML = FALSE)
# Add interaction between modality and trial
modXgame = lmer(trialLength.log ~ 1 + modalityCondition*condition +
             trialTotal + I(trialTotal^2) +
              condition:trialTotal + modalityCondition:trialTotal +
            matcherResponds +
            matcherResponds.cumulative +
            incorrect +
            multimodal +
            (1 + condition + incorrect |dyadNumber/playerId) +
            (1 + modalityCondition|itemId),
          data=d, REML = FALSE)

# Add 3-way interaction
moXcoXga =  lmer(trialLength.log ~ 1 + modalityCondition*condition*trialTotal +
             I(trialTotal^2) +
            matcherResponds +
            incorrect +
            multimodal +
            (1 + condition + incorrect |dyadNumber/playerId) +
            (1 + modalityCondition|itemId),
          data=d, REML = FALSE)
```

Interactions

```r
# interaction between turns and modality
nTurnXmo = lmer(trialLength.log ~ 1 + modalityCondition*condition*trialTotal +
             I(trialTotal^2) +
            matcherResponds + matcherResponds:modalityCondition +
            matcherResponds.cumulative +
            incorrect +
            multimodal +
            (1 + condition + incorrect |dyadNumber/playerId) +
            (1 + modalityCondition|itemId),
          data=d, REML = FALSE)

nTurnXco = lmer(trialLength.log ~ 1 + modalityCondition*condition*trialTotal +
             I(trialTotal^2) +
            matcherResponds + matcherResponds:modalityCondition +
            matcherResponds:condition +
            matcherResponds.cumulative +
            incorrect +
            multimodal +
            (1 + condition + incorrect |dyadNumber/playerId) +
```

```
            (1 + modalityCondition|itemId),
          data=d, REML = FALSE)

# Turn x modality x condtion
# Note that the acousitc modality had hardly any matcher turns,
# so the factor is dropped

tuXmoXco = lmer(trialLength.log ~ 1 + modalityCondition*condition*trialTotal +
             I(trialTotal^2) +
            matcherResponds*modalityCondition*condition +
            matcherResponds.cumulative +
            incorrect +
            multimodal +
            (1 + condition + incorrect |dyadNumber/playerId) +
            (1 + modalityCondition|itemId),
          data=d, REML = FALSE)
```

```
## fixed-effect model matrix is rank deficient so dropping 1 column / coefficient
```

```
# Add the interaction between modality and incorrectness
moXincor =   lmer(trialLength.log ~ 1 + modalityCondition*condition*trialTotal +
             I(trialTotal^2) +
            matcherResponds*modalityCondition*condition +
            matcherResponds.cumulative +
            incorrect + incorrect:modalityCondition +
            multimodal +
            (1 + condition + incorrect |dyadNumber/playerId) +
            (1 + modalityCondition|itemId),
          data=d, REML = FALSE)
```

```
## fixed-effect model matrix is rank deficient so dropping 1 column / coefficient
```

```
# Add the interaction between condition and incorrectness
coXincor =  lmer(trialLength.log ~ 1 + modalityCondition*condition*trialTotal +
             I(trialTotal^2) +
            matcherResponds*modalityCondition*condition +
            matcherResponds.cumulative +
            incorrect + incorrect:modalityCondition + incorrect:condition +
            multimodal +
            (1 + condition + incorrect |dyadNumber/playerId) +
            (1 + modalityCondition|itemId),
          data=d, REML = FALSE)
```

```
## fixed-effect model matrix is rank deficient so dropping 1 column / coefficient
```

```
# Add the three-way interaction between condition, modality and incorrectness
coXmoXin = lmer(trialLength.log ~ 1 + modalityCondition*condition*trialTotal +
             I(trialTotal^2) +
            matcherResponds*modalityCondition*condition +
            matcherResponds.cumulative +
            incorrect *modalityCondition*condition +
            multimodal +
            (1 + condition + incorrect |dyadNumber/playerId) +
            (1 + modalityCondition|itemId),
          data=d, REML = FALSE)
```

```
## fixed-effect model matrix is rank deficient so dropping 1 column / coefficient
```

```r
# Interaction between multimodality and condition
multiXco = lmer(trialLength.log ~ 1 + modalityCondition*condition*trialTotal +
             I(trialTotal^2) +
            matcherResponds*modalityCondition*condition +
            matcherResponds.cumulative +
            incorrect *modalityCondition*condition +
            multimodal + multimodal:condition +
            (1 + condition + incorrect |dyadNumber/playerId) +
            (1 + modalityCondition|itemId),
          data=d, REML = FALSE)
```

```
## fixed-effect model matrix is rank deficient so dropping 1 column / coefficient
```

```r
# Add interaction between quadratic effect of trial and modality
modXgamQ =  lmer(trialLength.log ~ 1 + modalityCondition*condition*trialTotal +
             I(trialTotal^2) +(modalityCondition:I(trialTotal^2)) +
            matcherResponds*modalityCondition*condition +
            matcherResponds.cumulative +
            incorrect *modalityCondition*condition +
            multimodal + multimodal:condition +
            (1 + condition + incorrect |dyadNumber/playerId) +
            (1 + modalityCondition|itemId),
          data=d, REML = FALSE)
```

```
## fixed-effect model matrix is rank deficient so dropping 1 column / coefficient
```

Interactions with matcher turns

```r
tMaTxMod = lmer(trialLength.log ~ 1 + modalityCondition*condition*trialTotal +
             I(trialTotal^2) +(modalityCondition:I(trialTotal^2)) +
            matcherResponds*modalityCondition*condition +
            matcherResponds.cumulative +
              matcherResponds.cumulative:modalityCondition +
            incorrect *modalityCondition*condition +
            multimodal + multimodal:condition +
            (1 + condition + incorrect |dyadNumber/playerId) +
            (1 + modalityCondition|itemId),
          data=d, REML = FALSE)
```

```
## fixed-effect model matrix is rank deficient so dropping 1 column / coefficient
```

Check block has no effect

```r
# Add block order
block =  lmer(trialLength.log ~ 1 + modalityCondition*condition*trialTotal +
             I(trialTotal^2) +(modalityCondition:I(trialTotal^2)) +
            matcherResponds*modalityCondition*condition +
            matcherResponds.cumulative +
              matcherResponds.cumulative:modalityCondition +
            incorrect *modalityCondition*condition +
            multimodal + multimodal:condition +
            matcherResponds +
            firstBlock +
            (1 + condition + incorrect |dyadNumber/playerId) +
            (1 + modalityCondition|itemId),
          data=d, REML = TRUE)# Last model is REML to get estimates
```

```
## fixed-effect model matrix is rank deficient so dropping 1 column / coefficient
```

```
# Add interaction between block order and modality
blocXmod = lmer(trialLength.log ~ 1 + modalityCondition*condition*trialTotal +
             I(trialTotal^2) +(modalityCondition:I(trialTotal^2)) +
            matcherResponds*modalityCondition*condition +
            matcherResponds.cumulative +
              matcherResponds.cumulative:modalityCondition +
            incorrect *modalityCondition*condition +
            multimodal + multimodal:condition +
            matcherResponds +
            firstBlock*modalityCondition +
            (1 + condition + incorrect |dyadNumber/playerId) +
            (1 + modalityCondition|itemId),
          data=d, REML = TRUE)
```

```
## fixed-effect model matrix is rank deficient so dropping 1 column / coefficient
```

Results

Compare the fit of the models:

```
modelComparison = anova(m0,modality,cond,game,modXcond,conXgame, modXgame,
      moXcoXga,mtchTrn,tMtchTr,tMaTxMod,nTurnXmo,nTurnXco,tuXmoXco,
      incor,moXincor,coXincor,coXmoXin,
      multim,multiXco,
      gamQuad,modXgamQ,
      block, blocXmod)
```

```
## refitting model(s) with ML (instead of REML)
```

```
attributes(modelComparison)$heading = ""
modelComparison
```

```
##
##          Df    AIC    BIC   logLik deviance    Chisq Chi Df Pr(>Chisq)
## m0       20 2686.0 2796.8 -1323.01   2646.0
## mtchTrn  21 2181.3 2297.6 -1069.64   2139.3 506.7419      1  < 2.2e-16 ***
## tMtchTr  22 2053.1 2174.9 -1004.53   2009.1 130.2089      1  < 2.2e-16 ***
## incor    23 2037.3 2164.7  -995.64   1991.3  17.7762      1  2.485e-05 ***
## multim   24 2038.2 2171.2  -995.12   1990.2   1.0451      1  0.3066294
## game     25 1761.1 1899.6  -855.54   1711.1 279.1529      1  < 2.2e-16 ***
## gamQuad  26 1712.8 1856.9  -830.41   1660.8  50.2634      1  1.344e-12 ***
## modality 28 1716.2 1871.3  -830.10   1660.2   0.6327      2  0.7287886
## cond     29 1717.7 1878.3  -829.83   1659.7   0.5376      1  0.4634404
## modXcond 31 1706.2 1877.9  -822.10   1644.2  15.4611      2  0.0004392 ***
## conXgame 32 1708.0 1885.3  -822.00   1644.0   0.1885      1  0.6641504
## modXgame 34 1701.5 1889.9  -816.75   1633.5  10.5073      2  0.0052284 **
## moXcoXga 35 1702.9 1896.8  -816.44   1632.9   0.6220      1  0.4303010
## nTurnXmo 38 1706.0 1916.5  -814.98   1630.0   2.9249      3  0.4033423
## nTurnXco 39 1707.8 1923.9  -814.90   1629.8   0.1425      1  0.7058457
## tuXmoXco 40 1709.0 1930.6  -814.48   1629.0   0.8569      1  0.3545994
## moXincor 42 1707.8 1940.5  -811.89   1623.8   5.1708      2  0.0753659 .
## coXincor 43 1709.7 1947.9  -811.86   1623.7   0.0630      1  0.8017564
## coXmoXin 45 1711.5 1960.8  -810.77   1621.5   2.1705      2  0.3378165
## multiXco 46 1713.2 1968.0  -810.58   1621.2   0.3966      1  0.5288414
## modXgamQ 48 1710.3 1976.3  -807.17   1614.3   6.8134      2  0.0331502 *
## tMaTxMod 50 1712.5 1989.5  -806.24   1612.5   1.8515      2  0.3962261
## block    51 1714.1 1996.6  -806.03   1612.1   0.4164      1  0.5187396
## blocXmod 53 1717.2 2010.9  -805.62   1611.2   0.8318      2  0.6597472
## ---
## Signif. codes:  0 '***' 0.001 '**' 0.01 '*' 0.05 '.' 0.1 ' ' 1
```

Pick final model for estimates:

```
finalModel = block
```

Final model estimates:

```
summary(finalModel)
```

```
## Linear mixed model fit by REML ['lmerMod']
## Formula:
## trialLength.log ~ 1 + modalityCondition * condition * trialTotal +
##     I(trialTotal^2) + (modalityCondition:I(trialTotal^2)) + matcherResponds *
```

```
##     modalityCondition * condition + matcherResponds.cumulative +
##     matcherResponds.cumulative:modalityCondition + incorrect *
##     modalityCondition * condition + multimodal + multimodal:condition +
##     matcherResponds + firstBlock + (1 + condition + incorrect |
##     dyadNumber/playerId) + (1 + modalityCondition | itemId)
##    Data: d
##
## REML criterion at convergence: 1744.1
##
## Scaled residuals:
##     Min      1Q  Median      3Q     Max
## -3.4137 -0.6137 -0.0552  0.5712  5.7083
##
## Random effects:
##  Groups              Name                     Variance Std.Dev. Corr
##  playerId:dyadNumber (Intercept)              0.043220 0.20790
##                      conditionVisual          0.028599 0.16911  -0.58
##                      incorrectTRUE            0.015801 0.12570  -0.72  0.16
##  itemId              (Intercept)              0.025369 0.15928
##                      modalityConditionvisual 0.002625 0.05123   0.80
##                      modalityConditionvocal  0.012299 0.11090  -0.09  0.52
##  dyadNumber          (Intercept)              0.066131 0.25716
##                      conditionVisual          0.023988 0.15488  -0.13
##                      incorrectTRUE            0.001567 0.03959  -0.46 -0.82
##  Residual                                     0.123399 0.35128
## Number of obs: 1882, groups:
## playerId:dyadNumber, 30; itemId, 16; dyadNumber, 15
##
## Fixed effects:
##                                                          Estimate
## (Intercept)                                             -0.530835
## modalityConditionvisual                                  0.498797
## modalityConditionvocal                                   0.378782
## conditionVisual                                          0.409872
## trialTotal                                              -0.157898
## I(trialTotal^2)                                          0.061550
## matcherRespondsTRUE                                      0.907786
## matcherResponds.cumulative                              -0.019613
## incorrectTRUE                                            0.268012
## multimodalTRUE                                           0.115162
## firstBlockVisual                                        -0.079581
## modalityConditionvisual:conditionVisual                 -0.247559
## modalityConditionvocal:conditionVisual                  -0.690612
## modalityConditionvisual:trialTotal                       0.018677
## modalityConditionvocal:trialTotal                        0.008403
## conditionVisual:trialTotal                              -0.001521
## modalityConditionvisual:I(trialTotal^2)                 -0.036022
## modalityConditionvocal:I(trialTotal^2)                  -0.002917
## modalityConditionvisual:matcherRespondsTRUE             -0.008214
## modalityConditionvocal:matcherRespondsTRUE              -0.105875
## conditionVisual:matcherRespondsTRUE                      0.088654
## modalityConditionvisual:matcherResponds.cumulative       0.021201
## modalityConditionvocal:matcherResponds.cumulative       -0.090406
## modalityConditionvisual:incorrectTRUE                   -0.077329
```

```
## modalityConditionvocal:incorrectTRUE                        -0.228144
## conditionVisual:incorrectTRUE                                0.027018
## conditionVisual:multimodalTRUE                              -0.064590
## modalityConditionvisual:conditionVisual:trialTotal           0.014131
## modalityConditionvocal:conditionVisual:trialTotal           -0.016712
## modalityConditionvisual:conditionVisual:matcherRespondsTRUE -0.104292
## modalityConditionvisual:conditionVisual:incorrectTRUE       -0.129780
## modalityConditionvocal:conditionVisual:incorrectTRUE         0.064822
##                                                             Std. Error
## (Intercept)                                                   0.173737
## modalityConditionvisual                                       0.197178
## modalityConditionvocal                                        0.498299
## conditionVisual                                               0.132324
## trialTotal                                                    0.019476
## I(trialTotal^2)                                               0.012668
## matcherRespondsTRUE                                           0.092247
## matcherResponds.cumulative                                    0.012612
## incorrectTRUE                                                 0.091586
## multimodalTRUE                                                0.058240
## firstBlockVisual                                              0.139013
## modalityConditionvisual:conditionVisual                       0.143128
## modalityConditionvocal:conditionVisual                        0.157212
## modalityConditionvisual:trialTotal                            0.028170
## modalityConditionvocal:trialTotal                             0.026254
## conditionVisual:trialTotal                                    0.025965
## modalityConditionvisual:I(trialTotal^2)                       0.017465
## modalityConditionvocal:I(trialTotal^2)                        0.017385
## modalityConditionvisual:matcherRespondsTRUE                   0.120640
## modalityConditionvocal:matcherRespondsTRUE                    0.374660
## conditionVisual:matcherRespondsTRUE                           0.116446
## modalityConditionvisual:matcherResponds.cumulative            0.016084
## modalityConditionvocal:matcherResponds.cumulative             0.212914
## modalityConditionvisual:incorrectTRUE                         0.121223
## modalityConditionvocal:incorrectTRUE                          0.125045
## conditionVisual:incorrectTRUE                                 0.101040
## conditionVisual:multimodalTRUE                                0.107341
## modalityConditionvisual:conditionVisual:trialTotal            0.036040
## modalityConditionvocal:conditionVisual:trialTotal             0.035938
## modalityConditionvisual:conditionVisual:matcherRespondsTRUE   0.156611
## modalityConditionvisual:conditionVisual:incorrectTRUE         0.137872
## modalityConditionvocal:conditionVisual:incorrectTRUE          0.133813
##                                                             t value
## (Intercept)                                                  -3.055
## modalityConditionvisual                                       2.530
## modalityConditionvocal                                        0.760
## conditionVisual                                               3.097
## trialTotal                                                   -8.107
## I(trialTotal^2)                                               4.859
## matcherRespondsTRUE                                           9.841
## matcherResponds.cumulative                                   -1.555
## incorrectTRUE                                                 2.926
## multimodalTRUE                                                1.977
## firstBlockVisual                                             -0.572
## modalityConditionvisual:conditionVisual                      -1.730
```

```
## modalityConditionvocal:conditionVisual                          -4.393
## modalityConditionvisual:trialTotal                                0.663
## modalityConditionvocal:trialTotal                                 0.320
## conditionVisual:trialTotal                                       -0.059
## modalityConditionvisual:I(trialTotal^2)                          -2.063
## modalityConditionvocal:I(trialTotal^2)                           -0.168
## modalityConditionvisual:matcherRespondsTRUE                      -0.068
## modalityConditionvocal:matcherRespondsTRUE                       -0.283
## conditionVisual:matcherRespondsTRUE                               0.761
## modalityConditionvisual:matcherResponds.cumulative                1.318
## modalityConditionvocal:matcherResponds.cumulative                -0.425
## modalityConditionvisual:incorrectTRUE                            -0.638
## modalityConditionvocal:incorrectTRUE                             -1.825
## conditionVisual:incorrectTRUE                                     0.267
## conditionVisual:multimodalTRUE                                   -0.602
## modalityConditionvisual:conditionVisual:trialTotal                0.392
## modalityConditionvocal:conditionVisual:trialTotal                -0.465
## modalityConditionvisual:conditionVisual:matcherRespondsTRUE  -0.666
## modalityConditionvisual:conditionVisual:incorrectTRUE            -0.941
## modalityConditionvocal:conditionVisual:incorrectTRUE              0.484
```

```
##
## Correlation matrix not shown by default, as p = 32 > 12.
## Use print(x, correlation=TRUE)  or
##   vcov(x)     if you need it
```

```
## fit warnings:
## fixed-effect model matrix is rank deficient so dropping 1 column / coefficient
```

Check model predictions. The model predictions are in the right range and direction, fitting linear quite well:

```
plot(predict(blocXmod),d$trialLength.log, pch=16, col=rgb(0,0,0,0.4),
     ylim=c(-1.5,2),xlim=c(-1.5,2))
abline(a=0,b=1, col=2, lwd=2)
abline(h=0, col=2)
abline(v=0, col=2)
```

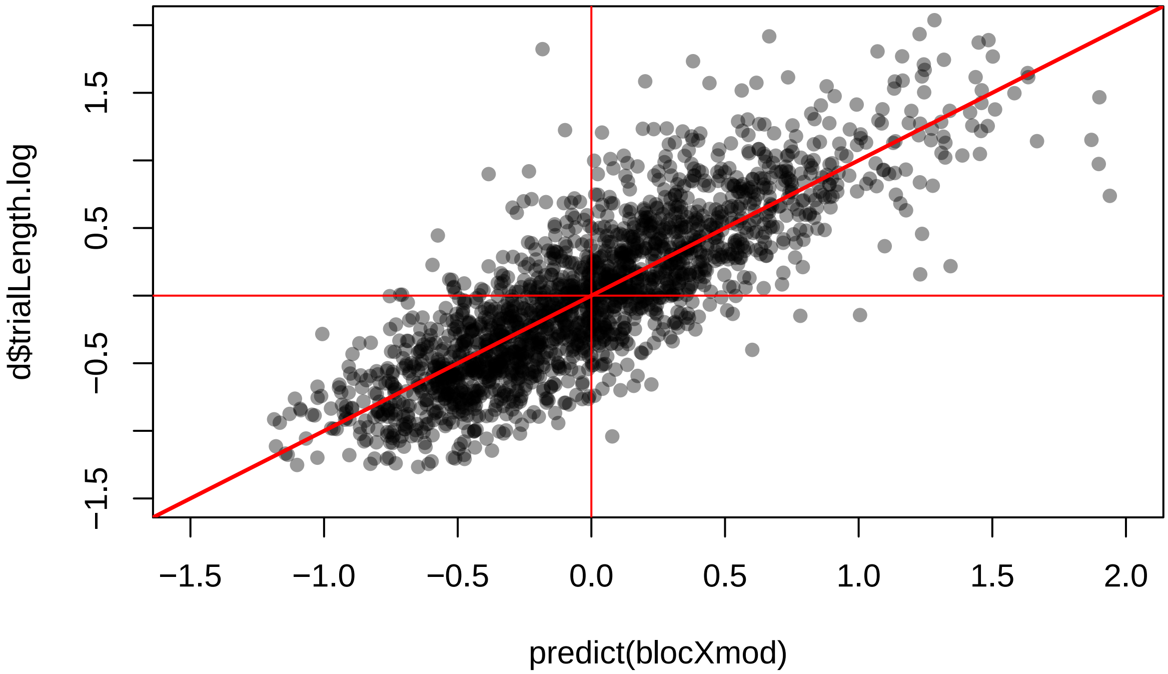


```
cor(predict(finalModel), d$trialLength.log)
```

```
## [1] 0.8336072
```

The residuals are ok, though it tends to do worse at higher values. This is expected from using the log scale.

```
qqnorm(resid(blocXmod))
qqline(resid(blocXmod))
```

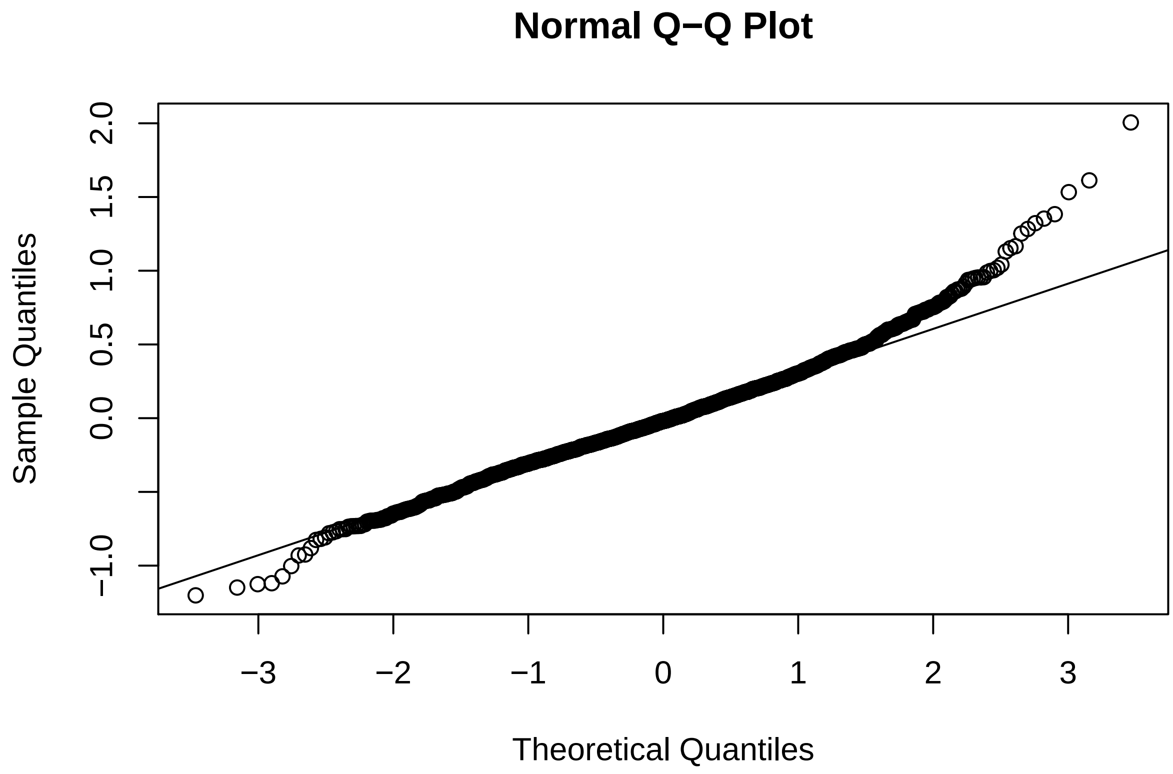

Plot the fixed effects

Relabel the effects:

```
feLabels = matrix(c(
"(Intercept)"              ,"Intercept"      , NA,
"modalityConditionvisual" ,"Visual modality", "modality",
"modalityConditionvocal"  , "Acoustic modality", "modality",
"conditionVisual" , "Visual stimuli","cond",
"trialTotal"              , "Game","game",
"modalityConditionvisual:conditionVisual"  , "Visual modality:Visual stimuli", "modXcond",
"modalityConditionvocal:conditionVisual" , "Acoustic modality:Visual stimuli","modXcond",
"modalityConditionvisual:trialTotal"    , "Visual modality:Game","modXgame",
"modalityConditionvocal:trialTotal"     , "Acoustic modality:Game", "modXgame",
"conditionVisual:trialTotal"            , "Visual stimuli:Game","conXgame",
"modalityConditionvisual:conditionVisual:trialTotal", "Visual modality:Visual stimuli:Game", "moXcoXga"
"modalityConditionvocal:conditionVisual:trialTotal", "Acoustic modality:Visual stimuli:Game", "moXcoXga
"incorrectTRUE","Incorrect","incor",
"modalityConditionvisual:incorrectTRUE","Visual modality:Incorrect","moXincor",
"modalityConditionvocal:incorrectTRUE","Acoustic modality:Incorrect","moXincor",
"modalityConditionvisual:I(trialTotal^2)", "Visual modality:Game^2","modXgamQ",
"modalityConditionvocal:I(trialTotal^2)", "Acoustic modality:Game^2","modXgamQ",
"I(trialTotal^2)","Game^2","gamQuad",
"firstBlockVisual","Visual stims first","block",
"modalityConditionvisual:firstBlockVisual","Visual modality:Visual stim first","blocXmod",
"modalityConditionvocal:firstBlockVisual","Acoustic modality:Visual stim first","blocXmod",
"conditionVisual:incorrectTRUE","Visual stimuli:incorrect","coXincor",
"modalityConditionvisual:conditionVisual:incorrectTRUE","Visual modality:Visual stimuli:incorrect","coXn
"modalityConditionvocal:conditionVisual:incorrectTRUE","Acoustic modality:Visual stimuli:incorrect","coX

"modalityConditionvisual:conditionVisual:numberOfTurns","VisualModality:Visual stim:NumTurns","tuXmoXco
"modalityConditionvisual:conditionVisual:matcherRespondsTRUE","VisualModality:Visual stim:Matcher Respon
"modalityConditionvocal:conditionVisual:numberOfTurns","Vocal Modality:Visual stim:NumTurns","tuXmoXco"
"modalityConditionvocal:conditionVisual:matcherRespondsTRUE","Vocal Modality:Visual stim:Matcher Respon
"conditionVisual:numberOfTurns","Visual stim:NumTurns","nTurnXco",
"conditionVisual:matcherRespondsTRUE","Visual stim:Matcher Responds","nTurnXco",
"modalityConditionvisual:numberOfTurns","VisualModality:NumTurns","nTurnXmo",
"modalityConditionvisual:matcherRespondsTRUE","VisualModality:Matcher Responds","nTurnXmo",
"modalityConditionvocal:numberOfTurns","Vocal Modality:NumTurns","nTurnXmo",
"modalityConditionvocal:matcherRespondsTRUE","Vocal Modality:Matcher Responds","nTurnXmo",
"numberOfTurns","Number of turns","nTurns",
"multimodalTRUE","Multimodal T1","multim",
"conditionVisual:multimodalTRUE","VisualStim:MultimodalT1","multiXco",
"matcherRespondsTRUE","Matcher Responds",'mtchTrn',
"matcherResponds.cumulative","Total interaction","tMtchTr",
"modalityConditionvisual:matcherResponds.cumulative","Total interaction:Visual Modality","tMaTxMod",
"modalityConditionvocal:matcherResponds.cumulative","Total interaction:Vocal Modality","tMaTxMod"
), ncol=3, byrow = T)

feLabels2 = as.vector(feLabels[match(names(fixef(finalModel)),feLabels[,1]),2])
feModel = as.vector(feLabels[match(names(fixef(finalModel)),feLabels[,1]),3])

sig = modelComparison$`Pr(>Chisq)`
names(sig) = rownames(modelComparison)
```

```r
sig.data = data.frame(estimate = fixef(finalModel),
                      y=1:length(fixef(finalModel)),
                      sig=sig[feModel])

cols= c("black",'red')
sig.data$pointCol = cols[1]
sig.data$pointCol[!is.na(sig.data$sig)] =
  cols[1 + (sig.data$sig[!is.na(sig.data$sig)] < 0.05)]
# Mark marginal effects
#sig.data$pointCol[!is.na(sig.data$sig) &
#                    sig.data$sig < 0.1 &
#                    sig.data$sig >=0.05] = "orange"

sig.data$fade = sig.data$sig > 0.05
```

Plot the strength of the fixed effects:

```r
x = sjp.lmer(finalModel, 'fe',
         show.intercept = T,
         sort.est=NULL,
         axis.labels = feLabels2[2:length(feLabels2)],
         xlab="Trial time (log ms)",
         geom.colors = c(1,1),
         show.p=F,
         show.values = F,
         p.kr = FALSE,
         string.interc="Intercept",
         prnt.plot = F)
```

```
## Computing p-values via Wald-statistics approximation (treating t as Wald z).
```

```r
x$plot.list[[1]]$data$fade = sig.data$fade

x$plot.list[[1]]
```

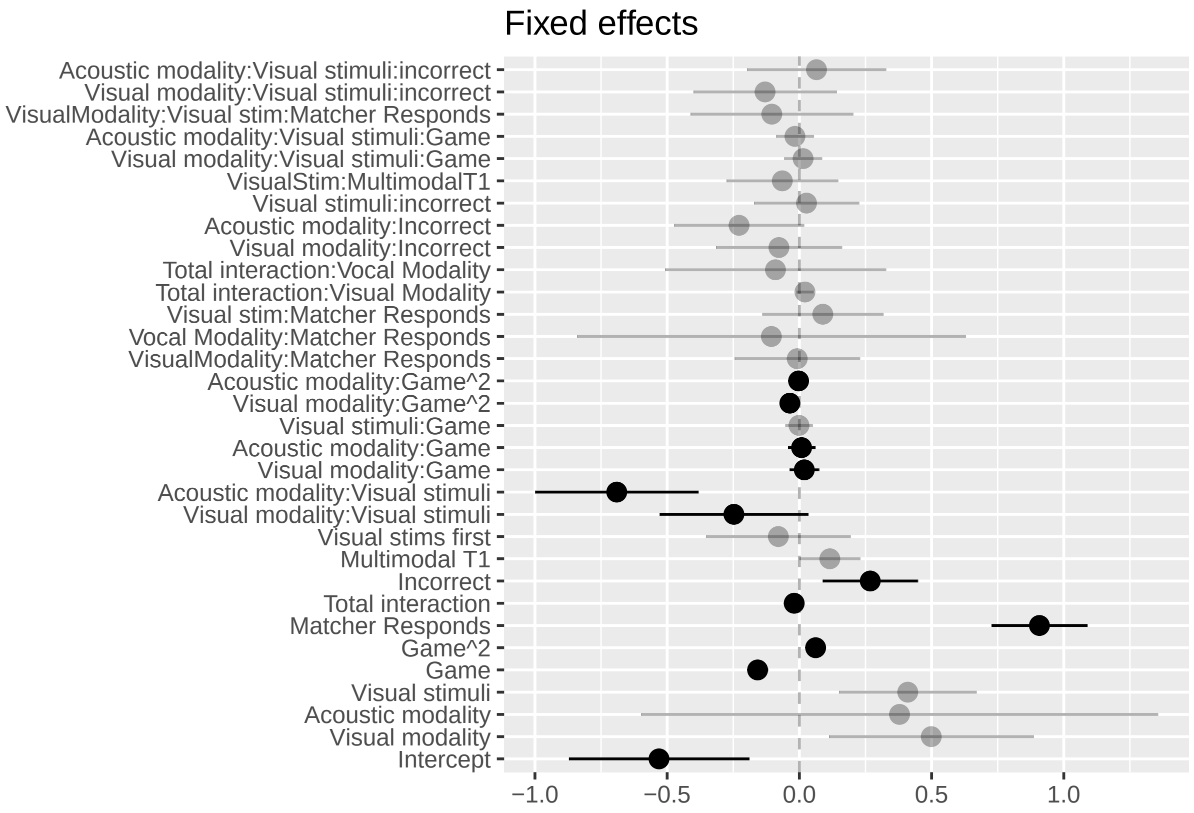
Fixed effects
Acoustic modality:Visual stimuli:incorrect
Visual modality:Visual stimuli:incorrect
VisualModality:Visual stim:Matcher Responds
Acoustic modality:Visual stimuli:Game
Visual modality:Visual stimuli:Game
VisualStim:MultimodalT1
Visual stimuli:incorrect
Acoustic modality:Incorrect
Visual modality:Incorrect
Total interaction:Vocal Modality
Total interaction:Visual Modality
Visual stim:Matcher Responds
Vocal Modality:Matcher Responds
VisualModality:Matcher Responds
Acoustic modality:Game^2
Visual modality:Game^2
Visual stimuli:Game
Acoustic modality:Game
Visual modality:Game
Acoustic modality:Visual stimuli
Visual modality:Visual stimuli
Visual stims first
Multimodal T1
Incorrect
Total interaction
Matcher Responds
Game^2
Game
Visual stimuli
Acoustic modality
Visual modality
Intercept
−1.0
−0.5
0.0
0.5
1.0

Attempt plot with axes in milliseconds.

```
convertEst = function(X){
  exp(meanLogTrialLength+X) - exp(meanLogTrialLength)
}

x$plot.list[[1]]$data$estimate =convertEst(x$plot.list[[1]]$data$estimate)
x$plot.list[[1]]$data$conf.low = convertEst(x$plot.list[[1]]$data$conf.low)
x$plot.list[[1]]$data$conf.high =  convertEst(x$plot.list[[1]]$data$conf.high)

sig.data2 = sig.data
sig.data2$estimate = x$plot.list[[1]]$data$estimate
sig.data2$estimate.lower = x$plot.list[[1]]$data$conf.low
sig.data2$estimate.upper = x$plot.list[[1]]$data$conf.high

x$plot.list[[1]]$data$fade = sig.data2$fade

x$plot.list[[1]] +
  scale_y_continuous(name="Difference (ms)") +
  scale_x_discrete(labels=feLabels2) +
  #geom_point(data=sig.data2,aes(y=estimate,x=y,fade=fade), color=sig.data$pointCol) +
  coord_flip(ylim=c(-5000,10000))
```

```
## Scale for 'x' is already present. Adding another scale for 'x', which
## will replace the existing scale.

## Coordinate system already present. Adding new coordinate system, which will replace the existing one
```

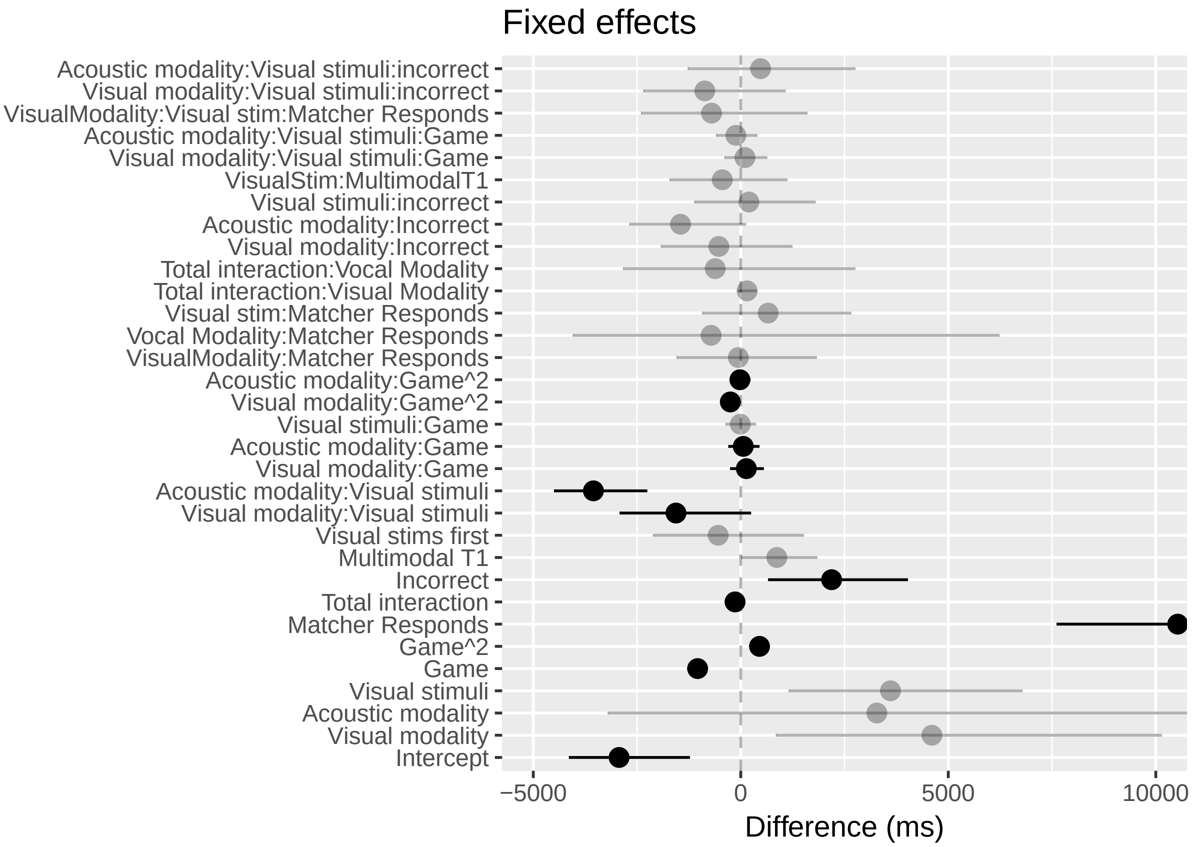

for every 10 trials where a matcher responded, subsequent trials were shorter by:

```
noInteraction = convertEst(
  fixef(finalModel)["(Intercept)"]
)

tenResponses = convertEst(
  fixef(finalModel)["(Intercept)"] +
  (10 * fixef(finalModel)["matcherResponds.cumulative"])
)

noInteraction - tenResponses
```

```
## (Intercept)
##    745.7498
```

Table for paper

```r
outdata = x$plot.list[[1]]$data[,c("estimate","conf.low",'conf.high')]

outdata$estimate = round(outdata$estimate)
outdata$conf.low = round(outdata$conf.low)
outdata$conf.high = round(outdata$conf.high)
#outdata = outdata[2:nrow(outdata),]

xd = as.data.frame(summary(finalModel)$coef)
#xd = xd[2:nrow(xd),]
outdata$wald.t = xd$`t value`

sig = modelComparison$`Pr(>Chisq)`
names(sig) = rownames(modelComparison)
sigx = sig[feModel]
#sigx = sigx[2:length(sigx)]

outdata$model.comparison.p = sigx
outdata$estimate = paste(
  c("","+")[1+(outdata$estimate>0)],
  as.character(outdata$estimate),sep='')

outdata$label = feLabels2

outdata = outdata[,c("label","estimate","conf.low",
                     "conf.high","wald.t",
                     "model.comparison.p")]

write.csv(outdata[2:nrow(outdata),],file="../../results/tables/Efficiency_FixedEffects.csv")
```

Random effects

There is a reasonable amount of variaition in the random effects, suggesting that dyads and players differ. This justifies the use of mixed effects modelling.

```r
dotplot(ranef(finalModel))
```

```
## $`playerId:dyadNumber`
```

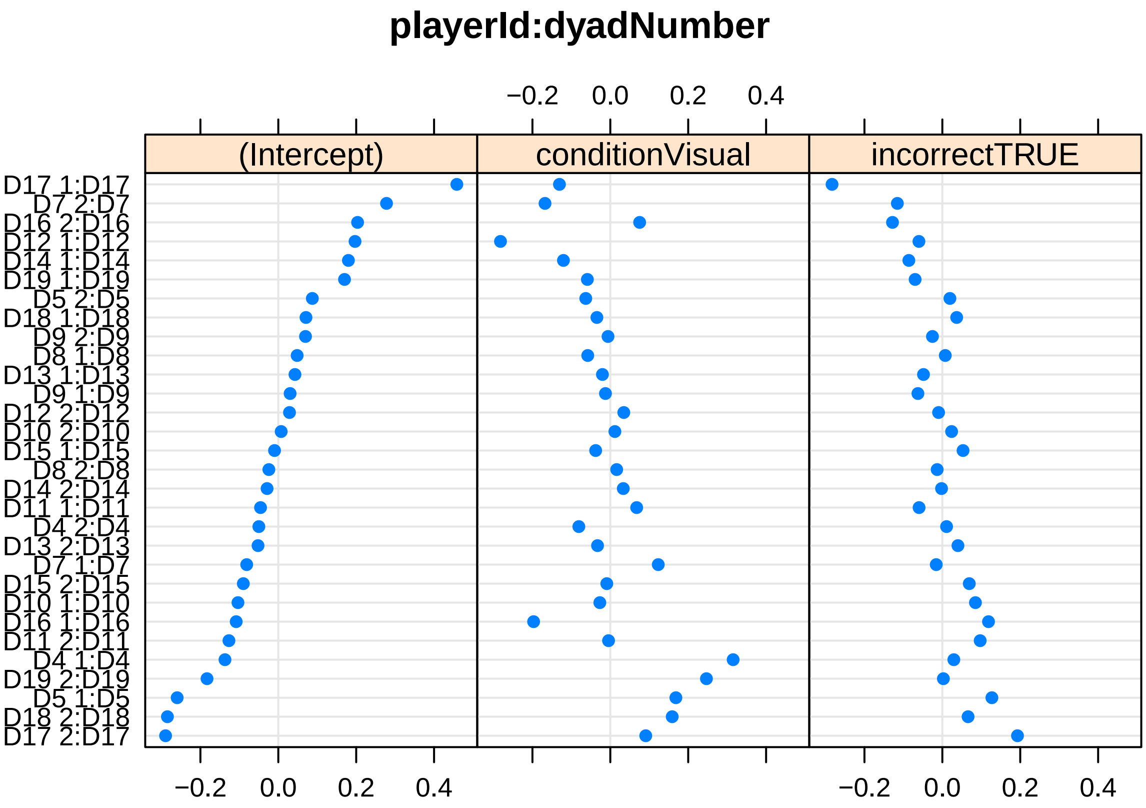
playerId:dyadNumber
(Intercept)
conditionVisual
incorrectTRUE
D17 1:D17
D7 2:D7
D16 2:D16
D12 1:D12
D14 1:D14
D19 1:D19
D5 2:D5
D18 1:D18
D9 2:D9
D8 1:D8
D13 1:D13
D9 1:D9
D12 2:D12
D10 2:D10
D15 1:D15
D8 2:D8
D14 2:D14
D11 1:D11
D4 2:D4
D13 2:D13
D7 1:D7
D15 2:D15
D10 1:D10
D16 1:D16
D11 2:D11
D4 1:D4
D19 2:D19
D5 1:D5
D18 2:D18
D17 2:D17
−0.2 0.0 0.2 0.4

```
##
## $itemId
```

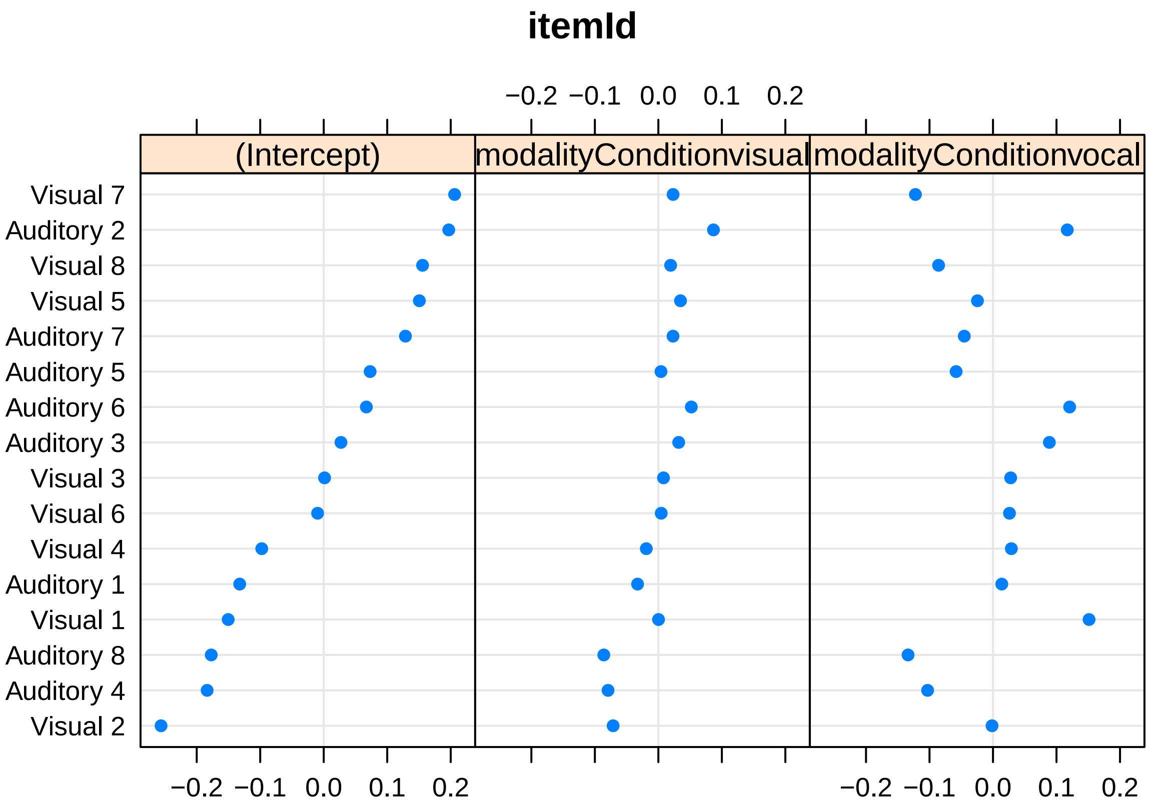
itemId
(Intercept)
modalityConditionvisual
modalityConditionvocal
Visual 7
Auditory 2
Visual 8
Visual 5
Auditory 7
Auditory 5
Auditory 6
Auditory 3
Visual 3
Visual 6
Visual 4
Auditory 1
Visual 1
Auditory 8
Auditory 4
Visual 2
−0.2 −0.1 0.0 0.1 0.2

```
##
```

```
## $dyadNumber
```

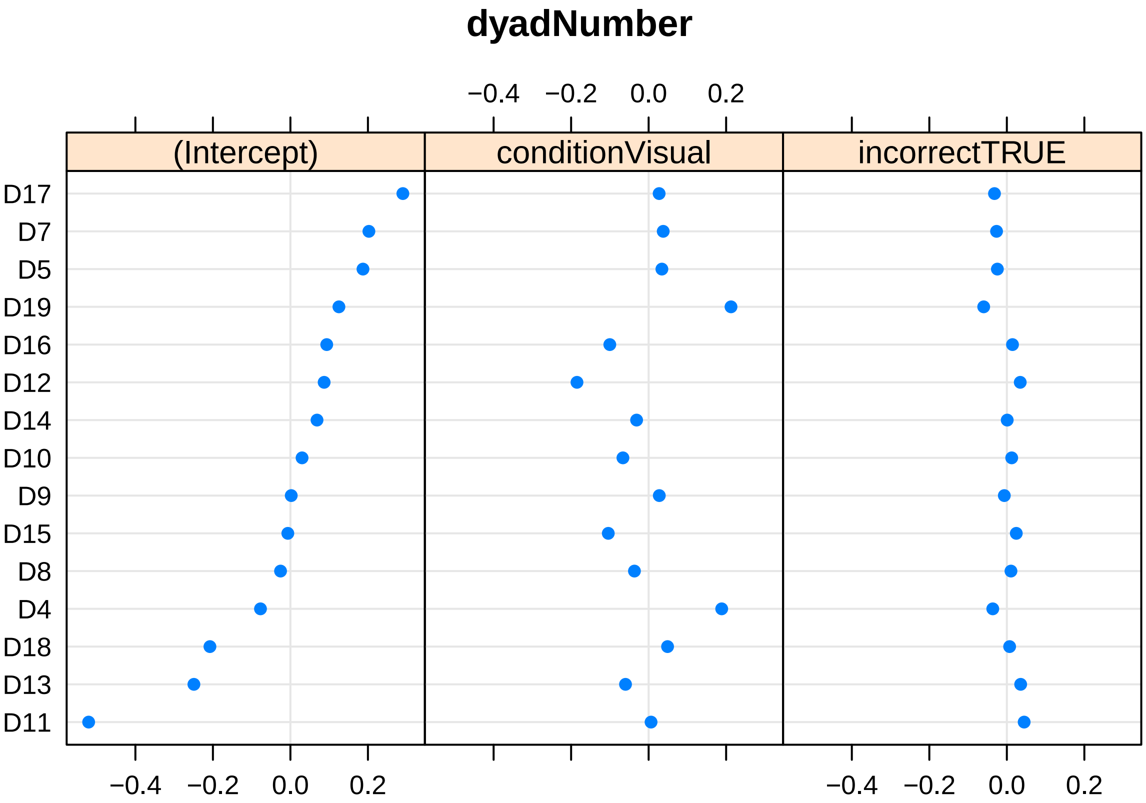

qq-plots of random effects

```r
sjp.lmer(finalModel, type = "re.qq")
```

```
## Testing for normal distribution. Dots should be plotted along the line.
```

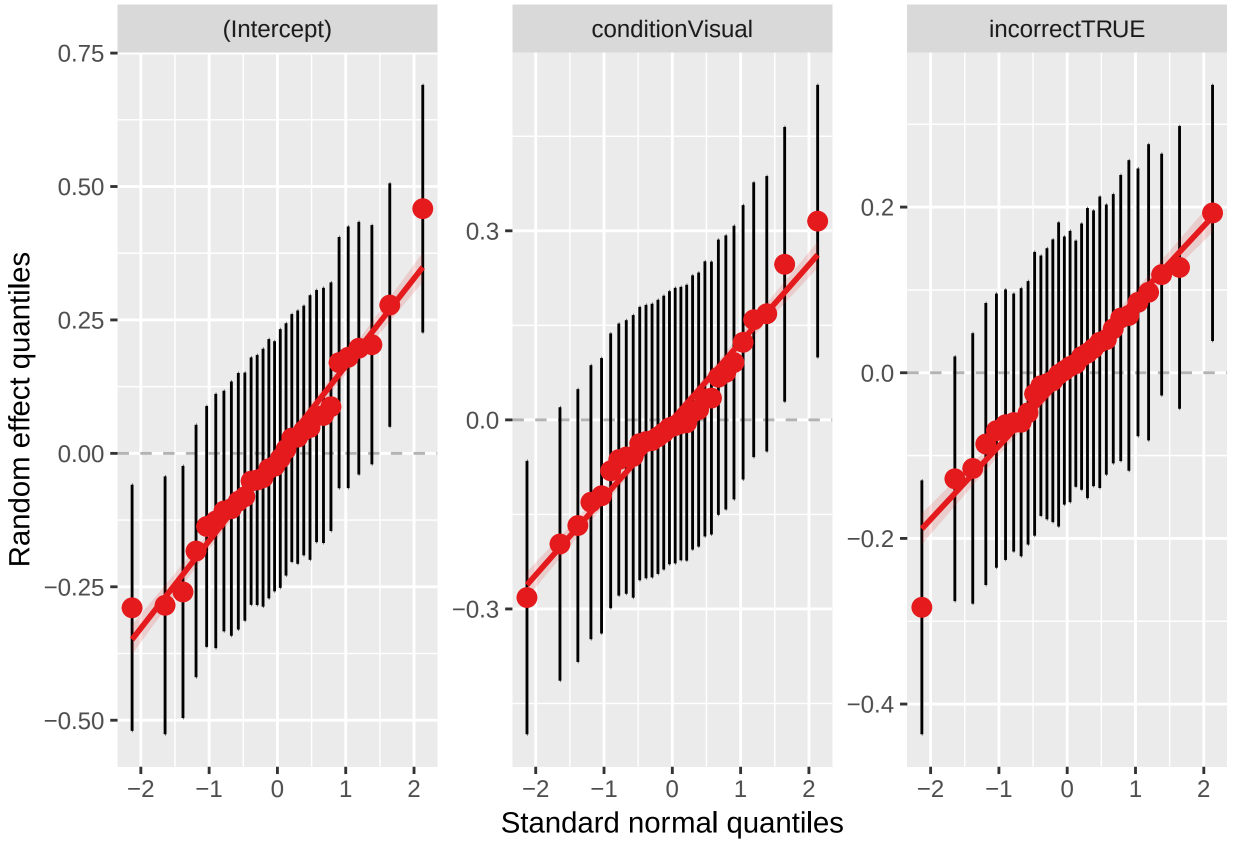

Relevel factors to see other comparisons

```
d2 = d
d2$condition = relevel(d2$condition,"Visual")
fm2 = update(finalModel, data=d2)
```

```
## fixed-effect model matrix is rank deficient so dropping 1 column / coefficient
```

```
summary(fm2)
```

```
## Linear mixed model fit by REML ['lmerMod']
## Formula:
## trialLength.log ~ 1 + modalityCondition * condition * trialTotal +
##     I(trialTotal^2) + (modalityCondition:I(trialTotal^2)) + matcherResponds *
##     modalityCondition * condition + matcherResponds.cumulative +
##     matcherResponds.cumulative:modalityCondition + incorrect *
##     modalityCondition * condition + multimodal + multimodal:condition +
##     matcherResponds + firstBlock + (1 + condition + incorrect |
##     dyadNumber/playerId) + (1 + modalityCondition | itemId)
##    Data: d2
##
## REML criterion at convergence: 1744.1
##
## Scaled residuals:
##     Min      1Q  Median      3Q     Max
## -3.4137 -0.6137 -0.0552  0.5712  5.7083
```

```
##
## Random effects:
##  Groups               Name                      Variance Std.Dev. Corr
##  playerId:dyadNumber (Intercept)               0.030839 0.17561
##                       conditionAuditory         0.028599 0.16911  -0.27
##                       incorrectTRUE             0.015801 0.12570  -0.70 -0.16
##  itemId               (Intercept)               0.025369 0.15928
##                       modalityConditionvisual   0.002625 0.05123   0.80
##                       modalityConditionvocal    0.012299 0.11090  -0.09  0.52
##  dyadNumber           (Intercept)               0.079952 0.28276
##                       conditionAuditory         0.023988 0.15488  -0.43
##                       incorrectTRUE             0.001567 0.03959  -0.87  0.82
##  Residual                                       0.123399 0.35128
## Number of obs: 1882, groups:
## playerId:dyadNumber, 30; itemId, 16; dyadNumber, 15
##
## Fixed effects:
##                                                             Estimate
## (Intercept)                                                -0.120964
## modalityConditionvisual                                     0.251238
## modalityConditionvocal                                     -0.311831
## conditionAuditory                                          -0.409872
## trialTotal                                                 -0.159418
## I(trialTotal^2)                                             0.061550
## matcherRespondsTRUE                                         0.996439
## matcherResponds.cumulative                                 -0.019613
## incorrectTRUE                                               0.295030
## multimodalTRUE                                              0.050572
## firstBlockVisual                                           -0.079581
## modalityConditionvisual:conditionAuditory                   0.247559
## modalityConditionvocal:conditionAuditory                    0.690612
## modalityConditionvisual:trialTotal                          0.032808
## modalityConditionvocal:trialTotal                          -0.008309
## conditionAuditory:trialTotal                                0.001521
## modalityConditionvisual:I(trialTotal^2)                    -0.036022
## modalityConditionvocal:I(trialTotal^2)                     -0.002917
## modalityConditionvisual:matcherRespondsTRUE                -0.112507
## modalityConditionvocal:matcherRespondsTRUE                 -0.105875
## conditionAuditory:matcherRespondsTRUE                      -0.088654
## modalityConditionvisual:matcherResponds.cumulative          0.021201
## modalityConditionvocal:matcherResponds.cumulative          -0.090407
## modalityConditionvisual:incorrectTRUE                      -0.207109
## modalityConditionvocal:incorrectTRUE                       -0.163323
## conditionAuditory:incorrectTRUE                            -0.027018
## conditionAuditory:multimodalTRUE                            0.064590
## modalityConditionvisual:conditionAuditory:trialTotal       -0.014131
## modalityConditionvocal:conditionAuditory:trialTotal         0.016712
## modalityConditionvisual:conditionAuditory:matcherRespondsTRUE  0.104292
## modalityConditionvisual:conditionAuditory:incorrectTRUE     0.129780
## modalityConditionvocal:conditionAuditory:incorrectTRUE     -0.064822
##                                                            Std. Error
## (Intercept)                                                  0.176530
## modalityConditionvisual                                      0.202962
## modalityConditionvocal                                       0.539640
```

```
## conditionAuditory                                                  0.132324
## trialTotal                                                         0.022960
## I(trialTotal^2)                                                    0.012668
## matcherRespondsTRUE                                                0.072111
## matcherResponds.cumulative                                         0.012612
## incorrectTRUE                                                      0.076437
## multimodalTRUE                                                     0.089998
## firstBlockVisual                                                   0.139012
## modalityConditionvisual:conditionAuditory                          0.143128
## modalityConditionvocal:conditionAuditory                           0.157212
## modalityConditionvisual:trialTotal                                 0.031605
## modalityConditionvocal:trialTotal                                  0.028902
## conditionAuditory:trialTotal                                       0.025965
## modalityConditionvisual:I(trialTotal^2)                            0.017465
## modalityConditionvocal:I(trialTotal^2)                             0.017385
## modalityConditionvisual:matcherRespondsTRUE                        0.101445
## modalityConditionvocal:matcherRespondsTRUE                         0.374660
## conditionAuditory:matcherRespondsTRUE                              0.116446
## modalityConditionvisual:matcherResponds.cumulative                 0.016084
## modalityConditionvocal:matcherResponds.cumulative                  0.212914
## modalityConditionvisual:incorrectTRUE                              0.109659
## modalityConditionvocal:incorrectTRUE                               0.100925
## conditionAuditory:incorrectTRUE                                    0.101040
## conditionAuditory:multimodalTRUE                                   0.107341
## modalityConditionvisual:conditionAuditory:trialTotal               0.036040
## modalityConditionvocal:conditionAuditory:trialTotal                0.035938
## modalityConditionvisual:conditionAuditory:matcherRespondsTRUE      0.156611
## modalityConditionvisual:conditionAuditory:incorrectTRUE            0.137872
## modalityConditionvocal:conditionAuditory:incorrectTRUE             0.133813
##                                                                  t value
## (Intercept)                                                       -0.685
## modalityConditionvisual                                            1.238
## modalityConditionvocal                                            -0.578
## conditionAuditory                                                 -3.097
## trialTotal                                                        -6.943
## I(trialTotal^2)                                                    4.859
## matcherRespondsTRUE                                               13.818
## matcherResponds.cumulative                                        -1.555
## incorrectTRUE                                                      3.860
## multimodalTRUE                                                     0.562
## firstBlockVisual                                                  -0.572
## modalityConditionvisual:conditionAuditory                          1.730
## modalityConditionvocal:conditionAuditory                           4.393
## modalityConditionvisual:trialTotal                                 1.038
## modalityConditionvocal:trialTotal                                 -0.287
## conditionAuditory:trialTotal                                       0.059
## modalityConditionvisual:I(trialTotal^2)                           -2.063
## modalityConditionvocal:I(trialTotal^2)                            -0.168
## modalityConditionvisual:matcherRespondsTRUE                       -1.109
## modalityConditionvocal:matcherRespondsTRUE                        -0.283
## conditionAuditory:matcherRespondsTRUE                             -0.761
## modalityConditionvisual:matcherResponds.cumulative                 1.318
## modalityConditionvocal:matcherResponds.cumulative                 -0.425
## modalityConditionvisual:incorrectTRUE                             -1.889
```

```
## modalityConditionvocal:incorrectTRUE                           -1.618
## conditionAuditory:incorrectTRUE                                -0.267
## conditionAuditory:multimodalTRUE                                0.602
## modalityConditionvisual:conditionAuditory:trialTotal           -0.392
## modalityConditionvocal:conditionAuditory:trialTotal             0.465
## modalityConditionvisual:conditionAuditory:matcherRespondsTRUE   0.666
## modalityConditionvisual:conditionAuditory:incorrectTRUE         0.941
## modalityConditionvocal:conditionAuditory:incorrectTRUE         -0.484
```

```
##
## Correlation matrix not shown by default, as p = 32 > 12.
## Use print(x, correlation=TRUE)  or
##   vcov(x)     if you need it
```

```
## fit warnings:
## fixed-effect model matrix is rank deficient so dropping 1 column / coefficient
```

```
feLabelsB = feLabels2
feLabelsB = gsub("Visual stimuli","Acoustic stimuli",feLabelsB)
feLabelsB = gsub("VisualStim","AcousticStim",feLabelsB)
feLabelsB = gsub("Visual stim","AcousticStim",feLabelsB)

x2 = sjp.lmer(fm2, 'fe',
         show.intercept = T,
         sort.est=NULL,
         axis.labels = feLabelsB[2:length(feLabelsB)],
         xlab="Trial time (log ms)",
         geom.colors = c(1,1),
         show.p=F,
         show.values = F,
         p.kr = FALSE,
         string.interc="Intercept",
         prnt.plot = F)
```

```
## Computing p-values via Wald-statistics approximation (treating t as Wald z).
```

```
x2$plot.list[[1]]$data$fade = sig.data$fade

x2$plot.list[[1]]
```

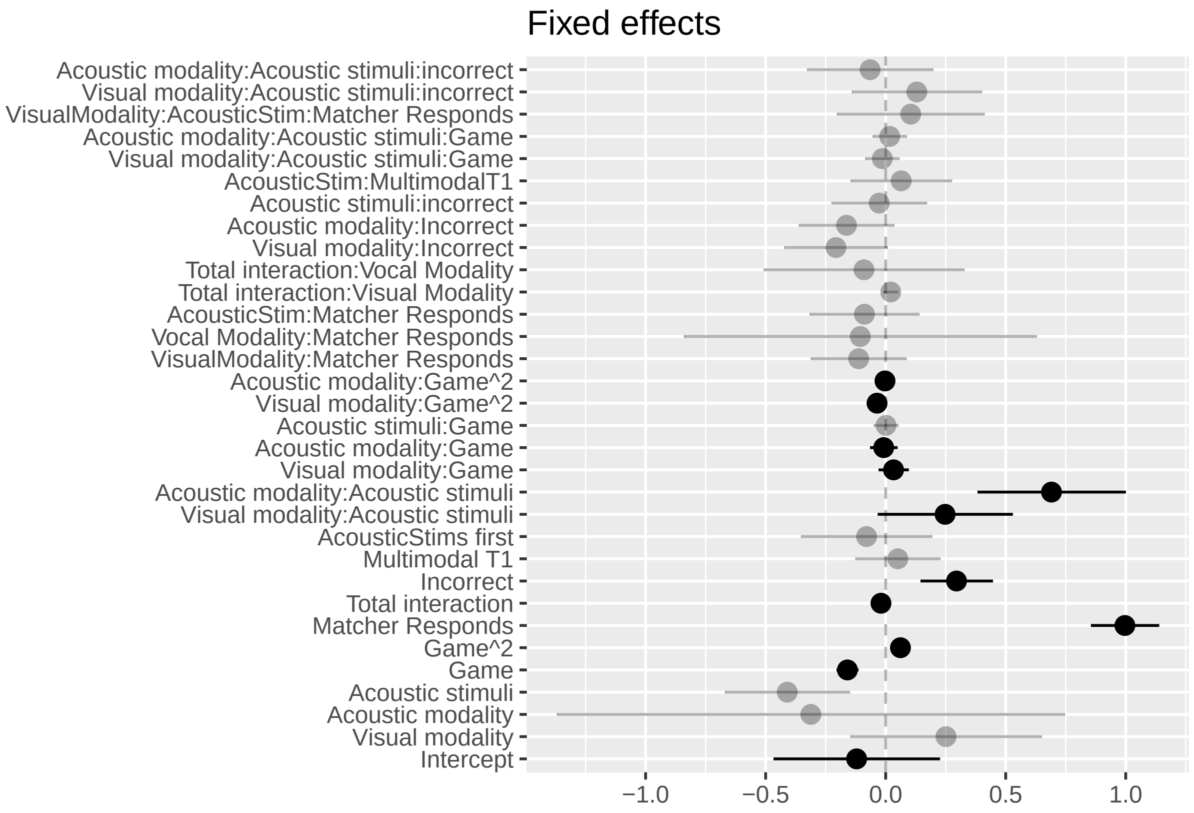


```
d2 = d
d2$modalityCondition = relevel(d2$modalityCondition,"visual")
fm2 = update(finalModel, data=d2)
```

```
## fixed-effect model matrix is rank deficient so dropping 1 column / coefficient
```

```
summary(fm2)
```

```
## Linear mixed model fit by REML ['lmerMod']
## Formula:
## trialLength.log ~ 1 + modalityCondition * condition * trialTotal +
##     I(trialTotal^2) + (modalityCondition:I(trialTotal^2)) + matcherResponds *
##     modalityCondition * condition + matcherResponds.cumulative +
##     matcherResponds.cumulative:modalityCondition + incorrect *
##     modalityCondition * condition + multimodal + multimodal:condition +
##     matcherResponds + firstBlock + (1 + condition + incorrect |
##     dyadNumber/playerId) + (1 + modalityCondition | itemId)
##    Data: d2
##
## REML criterion at convergence: 1744.1
##
## Scaled residuals:
##     Min      1Q  Median      3Q     Max
## -3.4137 -0.6137 -0.0552  0.5712  5.7083
##
## Random effects:
##  Groups              Name                    Variance Std.Dev. Corr
##  playerId:dyadNumber (Intercept)             0.043221 0.20790
##                      conditionVisual         0.028599 0.16911  -0.58
```

```
##                      incorrectTRUE         0.015801 0.12570  -0.72  0.16
##  itemId              (Intercept)           0.041101 0.20273
##                      modalityConditionmulti 0.002625 0.05123  -0.88
##                      modalityConditionvocal 0.009037 0.09507  -0.41 -0.07
##  dyadNumber          (Intercept)           0.066131 0.25716
##                      conditionVisual       0.023988 0.15488  -0.13
##                      incorrectTRUE         0.001567 0.03959  -0.46 -0.82
##  Residual                                  0.123399 0.35128
## Number of obs: 1882, groups:
## playerId:dyadNumber, 30; itemId, 16; dyadNumber, 15
##
## Fixed effects:
##                                                       Estimate
## (Intercept)                                          -0.032038
## modalityConditionmulti                               -0.498797
## modalityConditionvocal                               -0.120015
## conditionVisual                                       0.162312
## trialTotal                                           -0.139220
## I(trialTotal^2)                                       0.025529
## matcherRespondsTRUE                                   0.899571
## matcherResponds.cumulative                            0.001588
## incorrectTRUE                                         0.190682
## multimodalTRUE                                        0.115162
## firstBlockVisual                                     -0.079581
## modalityConditionmulti:conditionVisual                0.247559
## modalityConditionvocal:conditionVisual               -0.443053
## modalityConditionmulti:trialTotal                    -0.018677
## modalityConditionvocal:trialTotal                    -0.010274
## conditionVisual:trialTotal                            0.012610
## modalityConditionmulti:I(trialTotal^2)                0.036022
## modalityConditionvocal:I(trialTotal^2)                0.033105
## modalityConditionmulti:matcherRespondsTRUE            0.008214
## modalityConditionvocal:matcherRespondsTRUE           -0.097661
## conditionVisual:matcherRespondsTRUE                  -0.015639
## modalityConditionmulti:matcherResponds.cumulative    -0.021201
## modalityConditionvocal:matcherResponds.cumulative    -0.111606
## modalityConditionmulti:incorrectTRUE                  0.077329
## modalityConditionvocal:incorrectTRUE                 -0.150815
## conditionVisual:incorrectTRUE                        -0.102762
## conditionVisual:multimodalTRUE                       -0.064590
## modalityConditionmulti:conditionVisual:trialTotal    -0.014131
## modalityConditionvocal:conditionVisual:trialTotal    -0.030843
## modalityConditionmulti:conditionVisual:matcherRespondsTRUE  0.104292
## modalityConditionmulti:conditionVisual:incorrectTRUE       0.129780
## modalityConditionvocal:conditionVisual:incorrectTRUE       0.194602
##                                                      Std. Error
## (Intercept)                                            0.174969
## modalityConditionmulti                                 0.197178
## modalityConditionvocal                                 0.496076
## conditionVisual                                        0.137680
## trialTotal                                             0.020395
## I(trialTotal^2)                                        0.012021
## matcherRespondsTRUE                                    0.077698
## matcherResponds.cumulative                             0.010028
```

```
## incorrectTRUE                                                  0.080744
## multimodalTRUE                                                 0.058240
## firstBlockVisual                                               0.139012
## modalityConditionmulti:conditionVisual                         0.143128
## modalityConditionvocal:conditionVisual                         0.146377
## modalityConditionmulti:trialTotal                              0.028170
## modalityConditionvocal:trialTotal                              0.026928
## conditionVisual:trialTotal                                     0.025117
## modalityConditionmulti:I(trialTotal^2)                         0.017465
## modalityConditionvocal:I(trialTotal^2)                         0.016917
## modalityConditionmulti:matcherRespondsTRUE                     0.120640
## modalityConditionvocal:matcherRespondsTRUE                     0.371386
## conditionVisual:matcherRespondsTRUE                            0.104926
## modalityConditionmulti:matcherResponds.cumulative              0.016084
## modalityConditionvocal:matcherResponds.cumulative              0.212751
## modalityConditionmulti:incorrectTRUE                           0.121223
## modalityConditionvocal:incorrectTRUE                           0.117060
## conditionVisual:incorrectTRUE                                  0.095733
## conditionVisual:multimodalTRUE                                 0.107341
## modalityConditionmulti:conditionVisual:trialTotal              0.036040
## modalityConditionvocal:conditionVisual:trialTotal              0.035299
## modalityConditionmulti:conditionVisual:matcherRespondsTRUE     0.156611
## modalityConditionmulti:conditionVisual:incorrectTRUE           0.137873
## modalityConditionvocal:conditionVisual:incorrectTRUE           0.129480
##                                                              t value
## (Intercept)                                                   -0.183
## modalityConditionmulti                                        -2.530
## modalityConditionvocal                                        -0.242
## conditionVisual                                                1.179
## trialTotal                                                    -6.826
## I(trialTotal^2)                                                2.124
## matcherRespondsTRUE                                           11.578
## matcherResponds.cumulative                                     0.158
## incorrectTRUE                                                  2.362
## multimodalTRUE                                                 1.977
## firstBlockVisual                                              -0.572
## modalityConditionmulti:conditionVisual                         1.730
## modalityConditionvocal:conditionVisual                        -3.027
## modalityConditionmulti:trialTotal                             -0.663
## modalityConditionvocal:trialTotal                             -0.382
## conditionVisual:trialTotal                                     0.502
## modalityConditionmulti:I(trialTotal^2)                         2.063
## modalityConditionvocal:I(trialTotal^2)                         1.957
## modalityConditionmulti:matcherRespondsTRUE                     0.068
## modalityConditionvocal:matcherRespondsTRUE                    -0.263
## conditionVisual:matcherRespondsTRUE                           -0.149
## modalityConditionmulti:matcherResponds.cumulative             -1.318
## modalityConditionvocal:matcherResponds.cumulative             -0.525
## modalityConditionmulti:incorrectTRUE                           0.638
## modalityConditionvocal:incorrectTRUE                          -1.288
## conditionVisual:incorrectTRUE                                 -1.073
## conditionVisual:multimodalTRUE                                -0.602
## modalityConditionmulti:conditionVisual:trialTotal             -0.392
## modalityConditionvocal:conditionVisual:trialTotal             -0.874
```

```
## modalityConditionmulti:conditionVisual:matcherRespondsTRUE   0.666
## modalityConditionmulti:conditionVisual:incorrectTRUE         0.941
## modalityConditionvocal:conditionVisual:incorrectTRUE         1.503
##
## Correlation matrix not shown by default, as p = 32 > 12.
## Use print(x, correlation=TRUE)  or
##   vcov(x)     if you need it
## fit warnings:
## fixed-effect model matrix is rank deficient so dropping 1 column / coefficient
```

```
feLabelsB = feLabels2
feLabelsB = gsub("Visual modality","Multimodal",feLabelsB)


x2 = sjp.lmer(fm2, 'fe',
         show.intercept = T,
         sort.est=NULL,
         axis.labels = feLabelsB[2:length(feLabelsB)],
         xlab="Trial time (log ms)",
         geom.colors = c(1,1),
         show.p=F,
         show.values = F,
         p.kr = FALSE,
         string.interc="Intercept",
         prnt.plot = F)
```

```
## Computing p-values via Wald-statistics approximation (treating t as Wald z).
```

```
x2$plot.list[[1]]$data$fade = sig.data$fade

x2$plot.list[[1]]
```

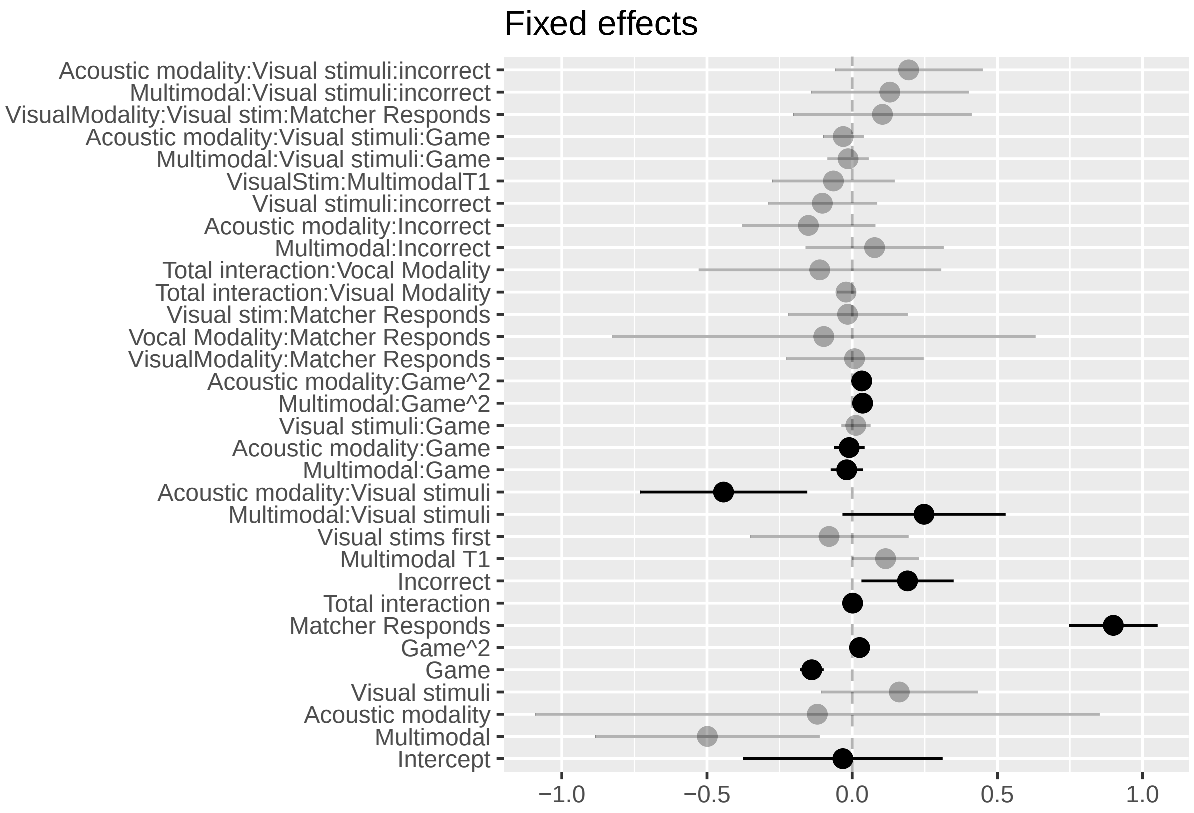

More plots

```
x = sjp.lmer(tMtchTr,'eff', 'matcherResponds.cumulative', show.ci = T, prnt.plot = F)$plot
```

```
x$data$y = exp(x$data$y + meanLogTrialLength)
x$data$ci.low = exp(x$data$lower + meanLogTrialLength)
x$data$ci.high = exp(x$data$upper + meanLogTrialLength)
x$data$x = x$data$x + matcherResponds.cumulative.mean

x$labels$title = NULL

pdf("../../results/graphs/CumulativeMatcherTurns_Efficiency.pdf",
    width=4,height=4)
x + #xlim(0,10) +
  geom_ribbon(aes(ymin=ci.low,ymax=ci.high),alpha = 0.15) +
  scale_x_continuous(limits=c(0,15)) +
  scale_y_continuous(breaks = c(0,3000,6000,9000,12000)) +
  #coord_cartesian(xlim = c(5, 10))  +
  xlab("Number of previous trials where\nmatcher responded") +
  ylab("Trial time (ms)") +
  theme(strip.background = element_blank(),
       strip.text.x = element_text(colour = 'white'))
dev.off()
```
